# UV damage induces production of mitochondrial DNA fragments with specific length profiles

**DOI:** 10.1093/genetics/iyae070

**Published:** 2024-05-09

**Authors:** Gus Waneka, Joseph Stewart, John R Anderson, Wentao Li, Jeffrey Wilusz, Juan Lucas Argueso, Daniel B Sloan

**Affiliations:** Department of Biology, Colorado State University, Fort Collins 80521, CO, USA; Department of Environmental and Radiological Health Sciences, Colorado State University, Fort Collins 80521, CO, USA; Cell and Molecular Biology Graduate Program, Colorado State University, Fort Collins 80521, CO, USA; Department of Microbiology, Immunology and Pathology, Colorado State University, Fort Collins 80521, CO, USA; Department of Environmental Health Science, University of Georgia, Athens 30602, GA, USA; Department of Microbiology, Immunology and Pathology, Colorado State University, Fort Collins 80521, CO, USA; Department of Environmental and Radiological Health Sciences, Colorado State University, Fort Collins 80521, CO, USA; Cell and Molecular Biology Graduate Program, Colorado State University, Fort Collins 80521, CO, USA; Department of Biology, Colorado State University, Fort Collins 80521, CO, USA; Cell and Molecular Biology Graduate Program, Colorado State University, Fort Collins 80521, CO, USA

**Keywords:** photodamage, mitochondrial genome (mtDNA), XR sequencing, *Saccharomyces cerevisiae*, *Arabidopsis thaliana*, cyclobutane pyrimidine dimer (CPD), nucleotide excision repair (NER), mtDNA degradation

## Abstract

UV light is a potent mutagen that induces bulky DNA damage in the form of cyclobutane pyrimidine dimers (CPDs). Photodamage and other bulky lesions occurring in nuclear genomes can be repaired through nucleotide excision repair (NER), where incisions on both sides of a damaged site precede the removal of a single-stranded oligonucleotide containing the damage. Mitochondrial genomes (mtDNAs) are also susceptible to damage from UV light, but current evidence suggests that the only way to eliminate bulky mtDNA damage is through mtDNA degradation. Damage-containing oligonucleotides excised during NER can be captured with antidamage antibodies and sequenced (XR-seq) to produce high-resolution maps of active repair locations following UV exposure. We analyzed previously published datasets from *Arabidopsis thaliana, Saccharomyces cerevisiae*, and *Drosophila melanogaster* to identify reads originating from the mtDNA (and plastid genome in *A. thaliana*). In *A. thaliana* and *S. cerevisiae*, the mtDNA-mapping reads have unique length distributions compared to the nuclear-mapping reads. The dominant fragment size was 26 nt in *S. cerevisiae* and 28 nt in *A. thaliana* with distinct secondary peaks occurring in regular intervals. These reads also show a nonrandom distribution of di-pyrimidines (the substrate for CPD formation) with TT enrichment at positions 7–8 of the reads. Therefore, UV damage to mtDNA appears to result in production of DNA fragments of characteristic lengths and positions relative to the damaged location. The mechanisms producing these fragments are unclear, but we hypothesize that they result from a previously uncharacterized DNA degradation pathway or repair mechanism in mitochondria.

## Teaser

UV damage in mitochondrial genomes has been historically difficult to study. One potential solution is excision repair sequencing (XR-seq), where UV damaged molecules are captured and directly sequenced. The authors analysis of the mitochondrial derived reads in numerous publicly available XR-seq datasets provides valuable insights into the fate of photodamage in mitochondrial genomes. They show that in diverse eukaryotes, mitochondrially derived DNA fragments of characteristic length and nucleotide composition are produced following UV exposure, possibly from mtDNA degradation or repair.

## Introduction

Mitochondria are vital organelles involved in energy production and cellular metabolism. Due to the endosymbiotic origins of mitochondria, they retain their own genomes that are replicated, repaired, and inherited independently of nuclear DNA (nucDNA). Mitochondrial genome (mtDNA) mutation rates show over a 4000-fold variation across eukaryotes (Wolfe *et al.* 1987; Drouin *et al.* 2008; Smith *et al.* 2012; Havird and Sloan 2016), which likely reflects a wide range of mtDNA replication and repair mechanisms. However, significant gaps in our understanding of mtDNA repair mechanisms still remain (Rong *et al.* 2021).

The existence of multiple mtDNA copies within a cell (St. John 2016) led to the hypothesis that DNA repair mechanisms might not be necessary because damaged mtDNA could be degraded without undergoing repair and undamaged mtDNA could act as a template for mtDNA synthesis (Clayton *et al.* 1974; Druzhyna *et al.* 2008). This idea was bolstered by the observation in metazoans that mtDNA mutation rates are much higher than nucDNA mutation rates (Wolfe *et al.* 1987) and the fact that mitochondria are an abundant source of DNA-damaging reactive oxygen species (Harman 1972; Murphy 2009). In subsequent decades, however, researchers have determined that mtDNA repair is an important component of mtDNA maintenance and have begun to work out the mechanisms of various mtDNA repair pathways (Saki and Prakash 2017).

With only one known exception (Muthye and Lavrov 2021), mtDNA repair enzymes are encoded in the nucDNA, translated in the cytosol, and targeted to the mitochondria (Mower *et al.* 2012; Zardoya 2020). In some cases, mtDNA repair pathways are highly similar to nucDNA repair pathways, often utilizing enzymatic machinery that is dual-targeted to the nucleus and the mitochondria (Kazak *et al.* 2012). For example, chemically modified mtDNA and nucDNA bases are both removed through base excision repair (BER), which is perhaps the most ubiquitous and best studied mtDNA repair pathway (Szczesny *et al.* 2008). In contrast, mtDNAs appear to lack canonical mismatch repair (MMR), the principal pathway for correcting mismatches that arise through erroneous base incorporation during DNA replication in nucDNA (Modrich 2006). Instead, various novel/noncanonical MMR pathways may fill this role, with a piecemeal, taxon-specific distribution. For example, the Y-box-binding protein YB-1 has been shown to play a role in mismatch elimination in human cell lines, primarily through mismatch recognition and binding (de Souza-Pinto *et al.* 2009). Meanwhile, plants appear to utilize a noncanonical MMR pathway reliant on homologous recombination (HR), facilitated by *MSH1* (Wu *et al.* 2020b).

Nucleotide excision repair (NER) is the major nucDNA repair pathway for bulky DNA damage, a broad class of lesions that occur on one strand of DNA and are characterized by the covalent attachment of large chemical moieties or compounds (Wood 1999). Diverse types of bulky lesions can result from the binding of various chemicals, metabolites, or environmental agents to DNA, leading to structural distortions and functional impairment. NER pathways have evolved independently in bacteria and eukaryotes, with distinct variations in the protein components and regulatory mechanisms. However, both systems follow the same general mechanism in which single-stranded incisions are made both upstream and downstream of a damaged site, followed by the removal of a damage-containing oligonucleotide ranging from ∼10to 13 (bacterial NER) or ∼23 to 30 (eukaryotic nuclear NER) nt in length. A polymerase fills the resulting gap using the opposite strand as a template, and ligation completes the NER process (Sancar 1996). As is the case for MMR, mtDNAs are thought to lack a conventional NER pathway. Because there are no known alternative pathways for repair of bulky DNA damage in mtDNAs, it is generally assumed that it leads to mtDNA degradation (Clayton *et al.* 1974; Prakash 1975; Kazak *et al.* 2012; Saki and Prakash 2017), but open questions remain regarding the molecular components of mtDNA degradation, how such degradation would be coordinated, and how new mtDNA molecules could be recovered (Sakamoto and Takami 2018; Zhao 2019; Zhao *et al.* 2023).

Degradation of damaged mtDNAs has been documented in metazoan and yeast cells in response to a variety of DNA-damaging agents including UV (Bess *et al.* 2012, 2013), acrolein (Wang *et al.* 2017), gamma irradiation (Dan *et al.* 2020), H_2_O_2_ (Shokolenko *et al.* 2016), and enzymatically induced double-stranded breaks (DSBs) (Moretton *et al.* 2017). The timelines of mtDNA degradation exhibit considerable variation depending on the organism, cell type, and DNA-damaging agents involved; however, it typically proceeds slowly (taking as long as 72 hours in some cases; Bess *et al.* 2012, 2013). MtDNA degradation is frequently associated with mitochondrial fission and mitochondrial-specific autophagy, known as mitophagy (Wang *et al.* 2017; Dan *et al.* 2020). Mitophagy increases during genotoxic stress, but it also occurs in unperturbed cells as part of normal mitochondrial turnover and cellular energetics (Urbina-Varela *et al.* 2020), and defects in mitophagy are associated with multiple human diseases (Springer and Macleod 2016; Doblado *et al.* 2021).

UV light is a potent mutagen capable of causing multiple bulky lesions, predominately in the form of cyclobutane pyrimidine dimers (CPDs; ∼80% occurrence) but also as pyrimidine–pyrimidone (6-4) photoproducts [(6-4)-PPs; ∼20% occurrence] (Emmerich *et al.* 2020). In addition to repair through NER, some organisms possess photolyases for the direct chemical reversal of photodamage. Photolyases are damage-specific, meaning a CPD photolyase can only repair CPDs and (6-4)PP photolyases can only repair (6-4)PPs. All photolyases use blue light as an energy source (Sancar 2003).

Although photolyases are broadly distributed across the tree of life, they are not ubiquitous. Roughly half of bacteria, a quarter of archaea, most plants and fungi, and most vertebrates possess CPD photolyases; (6-4)PP photolyases are generally not as common (Goosen and Moolenaar 2008; Lucas-Lledó and Lynch 2009; Mei and Dvornyk 2015). Photolyases have also been shown to repair photodamage in mtDNAs of some plants (Takahashi *et al.* 2011) and some fungi (Prakash 1975; Yasui *et al.* 1992). For other groups, such as mammals, there is no known mechanism for the repair of photodamage in mtDNA.

A handful of studies aimed at detecting NER in mtDNA have yielded negative results (Clayton *et al.* 1974; Waters and Moustacchi 1974; Prakash 1975; Ledoux *et al.* 1992; Hunter *et al.* 2010; Takahashi *et al.* 2011). The earliest experiments leveraged the CPD nicking T4 endonuclease V to measure the amount of CPDs in mtDNAs of UV-exposed cells. Irradiated mammalian cells given time for dark repair (NER) or light repair (photolyase) showed the same amount of mtDNA CPDs as irradiated cells given no time for repair, suggesting there is a complete lack of photodamage repair in mammalian mtDNA (Clayton *et al.* 1974). Similar studies found that the yeast *Saccharomyces cerevisiae* also lacks dark repair of CPDs in mtDNA but does exhibit light repair (Waters and Moustacchi 1974; Prakash 1975), and subsequent work established that a dual-targeted CPD photolyase protects both nuclear and mitochondrial DNA in *S. cerevisiae* (Yasui *et al.* 1992). Tests for NER in mtDNA using qPCR in rice (Takahashi *et al.* 2011) and zebrafish (Hunter *et al.* 2010) found no reduction in the number of polymerase-blocking lesions after irradiated organisms were given periods of dark repair. qPCR studies with mice cells did detect a decrease in frequency of polymerase-blocking lesions in mtDNA after long periods of repair (8–24 hours), but this was attributed to the repair of nonpyrimidine dimer polymerase-blocking lesions, which can also be induced through UV irradiation (Kalinowski *et al.* 1992). It therefore remains unclear whether and how eukaryotes repair pyrimidine dimers in mtDNA. While photolyases may fill this role for some eukaryotes, they are missing entirely in some groups (mammals) or are only partially represented, such as in *S. cerevisiae*, which lacks a photolyase for the repair of the (6-4)PPs (Sancar 2004).

In recent years, a series of DNA sequencing techniques leveraging antibodies that specifically recognize CPDs or (6-4)PPs have been developed to characterize pyrimidine dimer formation and repair on genome-wide scales (Hu *et al.* 2015, 2017; Mao *et al.* 2016; Alhegaili *et al.* 2019). One technique referred to as DDIP-seq (short for damaged DNA immunoprecipitation) uses antidamage antibodies to capture and sequence damage-containing molecules from samples of sonicated DNA (∼100–300 bp) (Amente *et al.* 2019). A DDIP-seq study with human HaCaT cells (keratinocyte cell line) and anti-CPD antibodies showed that CPD damage occurs at a high rate in mtDNA immediately following UV exposure. Surprisingly, after 24 hours allowing for repair, as much as 50% of the mtDNA damage had disappeared (Alhegaili *et al.* 2019), contrasting with previous reports documenting no CPD repair in mammalian mtDNA (Clayton *et al.* 1974; Ledoux *et al.* 1992). Antidamage antibodies can also be used to detect excision oligos directly in excision assays, where damage-containing oligos are captured with antidamage antibodies, 3′ radiolabeled, and visualized on high-density polyacrylamide gels (Hu *et al.* 2013).

**Fig. 1.**
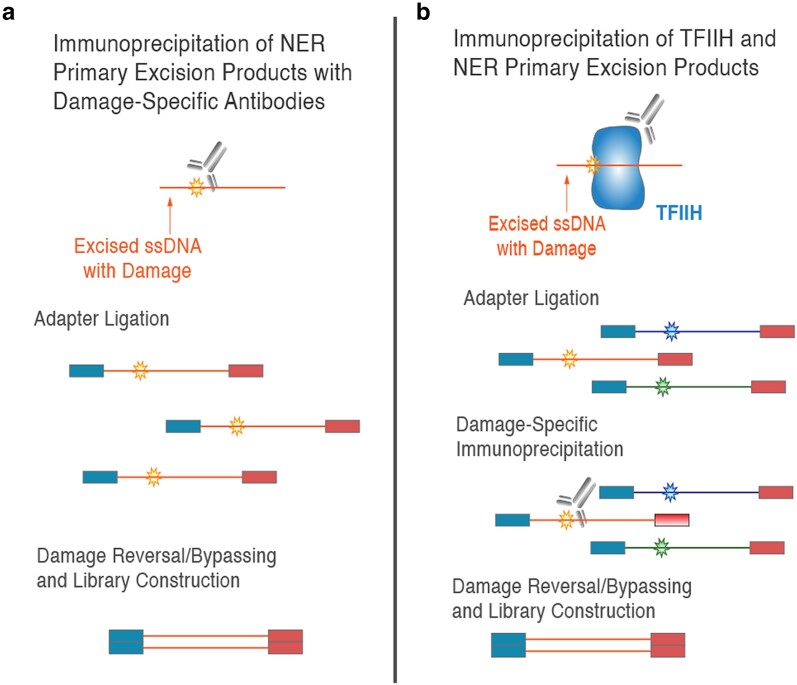
Overview of XR-seq protocol. Panel a shows direct capture of damage-containing excised oligomers as performed in experiments with *S. cerevisiae*, *A. thaliana*, and *D. melanogaster*. After immunoprecipitation with damage-specific antibodies, adaptors are attached to excised and the damaged sites are repaired by a photolyase before the molecules are amplified and sequenced. Panel b shows the alternative XR-seq approach, which includes an initial immunoprecipitation against transcription factor IIH (TFIIH; the enzymatic complex that associates with excised oligomers in mammalian cells). Then, adaptors are ligated to the ssDNA fragments before a second immunoprecipitation with antidamage antibodies, photolyase damage reversal, and library amplification/sequencing.

Another technique called XR-sequencing (XR-seq) has been particularly useful for understanding repair dynamics (Hu *et al.* 2015). XR-seq uses antidamage antibodies to capture the oligonucleotides that are excised during NER (Fig. 1a). These oligonucleotides are then subject to adaptor ligation, treated with photolyases, and sequenced on Illumina platforms. Sequenced reads can be aligned to reference genomes, yielding maps of active repair locations following UV exposure at single-nucleotide resolution. The technique achieves an extremely high sensitivity through the combined action of multiple filtering steps built into the library preparation (Hu *et al.* 2019). First, the antibodies have a high specificity for their damage targets (Mori *et al.* 1991), as evidenced by control immunoprecipitations with unirradiated cells, which yield no detectable DNA on polyacrylamide gels (Oztas *et al.* 2018). The anti-CPD and anti-(6-4)PP antibodies may bind damage in both ssDNA and dsDNA (Mori *et al.* 1991). However, dsDNAs containing CPDs should not receive adaptors, which anneal to ssDNA through overhanging, random 5-nt sequences. XR-seq experiments have been performed with cells or tissue samples from *Homo sapiens* (Hu *et al.* 2015), *Mus musculus* (Yang *et al.* 2018), *Microcebus murinus* (Akkose *et al.* 2021 ), *Drosophila melanogaster* (Deger *et al.* 2019), *S. cerevisiae* (Li *et al.* 2018), and *Arabidopsis thaliana* (Oztas *et al.* 2018). The mtDNA-mapping reads from these datasets remain largely unexplored.

It is possible that previous attempts to detect NER in mtDNA may have failed because of a relatively weak signal of mtDNA repair compared to dominant signal of NER in nucDNA (Zhao and Sumberaz 2020). We reasoned that the high sensitivity of XR-seq would provide increased power for detecting previously uncharacterized repair activity in mtDNA. If there is no repair pathway for excision of photodamage or other bulky DNA lesions in mtDNA (as is generally thought) and instead such lesions lead to mtDNA degradation and turnover, the XR-seq data can still provide valuable insights into fate of photodamage during degradation and whether degradation is ordered or localized to certain regions of the genome. Published mammalian XR-seq datasets are unsuitable for such mtDNA analysis because they include an initial immunoprecipitation against TFIIH, a nuclear-localized protein complex that associates with excised oligonucleotides in mammalian NER (Fig. 1b) (Lindsey-Boltz *et al.* 2023). Therefore, in this study, we analyzed the mtDNA-mapping reads from published *S. cerevisiae*, *A. thaliana*, and *D. melanogaster* datasets, in which the extracted small DNA molecules were immediately immunoprecipitated with antidamage antibodies (anti-CPD or anti-(6-4)PP) without an initial TFIIH immunoprecipitation (Fig. 1a).

## Methods

### XR-seq datasets

The XR-seq datasets from *S. cerevisiae, A. thaliana*, and *D. melanogaster* were generated in previous experiments (Li *et al.* 2018; Oztas *et al.* 2018; Deger *et al.* 2019, respectively). The methods used to generate those datasets are briefly summarized here. In the *A. thaliana* experiment, plants were irradiated with 120 J/m^2^ ultraviolet-C (UVC) at eight different times (spaced 3 hours apart) throughout a 24-hour day–night cycle and given 30 minutes of “dark repair” time (Oztas *et al.* 2018). In the *S. cerevisiae* experiment, cells were grown to late log phase and then irradiated with 120 J/m^2^ UVC and given either 5, 20, or 60 minutes of “dark repair” time (Li *et al.* 2018). Previous studies in *S. cerevisiae* estimate that a dose of 120 J/m^2^ UVC would result in approximately 1 CPD per 3 kb of DNA (Li *et al.* 2014). In the *D. melanogaster* experiment, S2 cells (from the Drosophila Genomics Resource Center; DGRC) were grown to 25–80% confluence and then irradiated with 20 J/m^2^ UVC and given either 0.16, 0.5, 8, 16, or 24 hours of “dark repair” time (Deger *et al.* 2019).

In all three experiments, two biological replicates were included for each timepoint. The library preparation protocols were similar in all experiments, though there were differences in the methods of DNA extraction. Specifically, for *S. cerevisiae* and *D. melanogaster*, cells were disrupted through bead beating and the excised DNA was enriched by Hirt lysis, where salt is used to precipitate away the chromatin fraction of the cell lysate, and through G-50 column filtration, which further depletes the chromatin fraction (Li *et al.* 2018; Deger *et al.* 2019; Hu *et al.* 2019). For *A. thaliana*, whole leaves were frozen in liquid nitrogen and ground into a powder before they were vortexed with glass beads (Oztas *et al.* 2018). In all three preparations, DNA was extracted through ethanol precipitation and damage-containing products were immunoprecipitated with anti-CPD or anti-(6-4)PP antibodies. Adaptors were ligated onto the excised oligomers before a second immunoprecipitation was performed to further enrich damage-containing molecules (Li *et al.* 2018; Oztas *et al.* 2018; Deger *et al.* 2019; Hu *et al.* 2019). In all three preparations, the adaptor-ligated products were then treated with photolyases (either CPD- or (6-4)PP-specific, depending on the library) before the samples were amplified and sequenced using 50-nt single-read Illumina chemistry.

### Alignment

Raw XR-seq reads were downloaded from NCBI BioProjects (*A. thaliana*: PRJNA429185, *D. melanogaster*: PRJNA577587, and *S. cerevisiae*: PRJNA434118) via the SRA Toolkit fastq-dump command (version 2.8.0; Andrews 2010). Adaptor sequences (reported in original publications: Li *et al.* 2018; Oztas *et al.* 2018; Deger *et al.* 2019) were removed with cutadapt (version 1.18; Martin 2011) using the discard untrimmed reads option. Reads were aligned to reference genomes (*A. thaliana*: TAIR10, *D. melanogaster*: dm6_UCSC, and *S. cerevisiae*: sacCer3), which included the organellar genomes (*A. thaliana* mtDNA: NC_037304.1, *A. thaliana* plastid DNA (ptDNA): NC_000932.1, *D. melanogaster* mtDNA: NC_024511.2, and *S. cerevisiae* mtDNA: NC_001224.1) using bowtie2 (version 2.3.5; Langmead and Salzberg 2012) with the –phred33 flag (Oztas *et al.* 2018).

### Alignment filtering and XR-seq analysis

Nuclear insertions of mtDNA or ptDNA (termed NUMTs and NUPTs, respectively) warrant special consideration in this analysis because repair of organelle-derived nucDNA through conventional NER could result in the false mapping of XR-seq reads to organelle genomes. To ensure that reads mapping to the organelle genomes truly originated from the organelle genomes, we used samtools (version 1.9; Li *et al.* 2009) to discard reads with mapping quality (MAPQ) scores of less than 30, effectively removing all reads which map equally well to multiple locations. As a result of this filtering step, NUMTs/NUPTs that are correctly assembled in the nuclear reference (and any homologous sequences present in the assemblies) are “unmappable” to either copy (organellar or nuclear). The *A. thaliana* ptDNA contains a large, inverted repeat (∼26 kb). Since both copies of the repeat would be “unmappable” after filtering out reads with MAPQ scores of less than 30, we removed the second copy of the repeat (positions 128214–154478) from the reference genome and divided all read counts in the first copy of the repeat by two when calculating coverage statistics. A 641-kb NUMT on chromosome 2 of the *A. thaliana* reference genome contains more than an entire copy of the mtDNA (Fields *et al.* 2022), which introduces a potential bias as only the identical portions of the NUMT and the mtDNA will be “unmappable” using a MAPQ cutoff of 30. We therefore used a modified reference where the NUMT (positions 3239038–3509765 of chromosome 2) was manually removed. While interpreting the *A. thaliana* dataset, it is therefore important to remember that some mtDNA-mapping reads may be nuclear-derived. After MAPQ filtering, we used custom scripts to remove reads with mismatches (all scripts used in this study are available via https://github.com/dbsloan/mtDNA_UV_damage).

We used custom scripts to calculate the read length distributions, nucleotide frequencies, and di-pyrimidine frequencies of the mtDNA-mapping reads and compared them to equivalent analyses from the nuclear-mapping reads, which were previously reported (Li *et al.* 2018; Oztas *et al.* 2018; Deger *et al.* 2019). We analyzed the differences in read coverage [reads per kilobase per million mapped reads (RPKM)] between organellar and nuclear genomes and between different genomic regions (i.e. intergenic, intronic, protein coding (CDS), rRNA genes, and tRNA genes) of the organellar genomes. In genic regions, we compared the XR-seq read coverage of the template vs the coding strand.

### Excision assay

To study mtDNA-derived DNA fragments with a method independent of the XR-seq data, we performed an excision assay with *S. cerevisiae* cells exposed to UV light. To isolate mtDNA-derived DNA fragments, we produced a NER-deficient line, which in theory should be unable to produce nucDNA-derived excision oligonucleotides. Specifically, we created a deletion of the *RAD14* gene, which encodes a subunit of nucleotide excision repair factor 1 (NEF1) complex that binds to damaged DNA during NER (Guzder *et al.* 2006). Deletions were generated through HR-mediated integration of the *NatMX4* nourseothricin resistance cassette (Goldstein and McCusker 1999) in strain FY86 (*MATα, ura3-52, leu2Δ1, his3Δ200*; Winston *et al.* 1995), which is isogenic with the S288c reference genome background. We amplified *NatMX4* from pAG25 using primers JAO2397 and JAO2398 (reported in Supplementary Table 1) to generate a PCR product flanked by 42-bp homologous regions (upper case in primer sequences), targeting integration to each side of the *RAD14* open reading frame. We screened transformants and confirmed the presence of the *rad14Δ::NatMX4* deletion in two independently generated clones, using PCR with primers flanking both sides of the insertion site (primers JAO2399 and JAO2401, reported in Supplementary Table 1).

Yeast growth, UV exposure, DNA extraction, immunoprecipitation with an anti-CPD antibody, radiolabeling, and DNA visualization all followed previously described protocols (Hu *et al.* 2019), with these exceptions: (1) UV exposure was performed in a CL-1000 UV crosslinker, which was placed on a shake plate rotating at 120 rpm to ensure even UV administration, (2) we radiolabeled the 3′ ends of the putative damaging containing DNA fragments with GTP [α-^32^P] (Boulé *et al.* 2001) instead of ^32^P-cordycepin due to changes in product availability, and (3) we added 5% glycerol to the 11% acrylamide gel mix and electrophoresis running buffering solutions in an attempt to reduce gel shattering while drying at 80°C (Altschuler *et al.* 2013). Following UV exposure, all work was conducted in the dark or under yellow light to avoid the activation of photolyases. We included wild type (WT) and *rad14Δ* replicates that were not exposed to UV as controls, and UV-exposed strains were given 20 minutes of repair time in YPD at 30°C. For each of the four treatments (WT vs mutant with or without UV exposure), we included two technical replicates for a total of eight samples.

## Results and discussion

### Preprocessing of existing XR-seq datasets

We analyzed the mtDNA-mapping reads from *S. cerevisiae*, *A. thaliana*, and *D. melanogaster* XR-seq datasets to gain insights into what happens to photodamaged mtDNA. In the *A. thaliana* dataset, we also investigated ptDNA-derived reads. Due to the short length of excised oligonucleotides in NER, nuclear-derived XR-seq sequences may map incorrectly to organellar genomes during alignment. To ensure such mapping artifacts are not interpreted as organellar-derived DNA fragments, we filtered our alignments to retain only uniquely mapping reads with no mismatches. We assessed the impact of this filtering step by comparing XR-seq coverage of the filtered and unfiltered alignment files and found that filtering renders 5–13% of organellar genomes “unmappable”. The fraction of each genome retained for downstream analyses, broken down by genomic region, is listed in Table 1.

**Table 1.** Fraction of each organellar genome retained after filtering to remove multimapping reads.

	*S. cerevisiae* mtDNA: CPD	*S. cerevisiae* mtDNA: (6-4)PP	*A. thaliana* mtDNA*^a^*: CPD	*A. thaliana* ptDNA*^b^*: CPD	*D. melanogaster* mtDNA: CPD
Intergenic	0.88	0.88	0.88	0.95	0.49
Intron	0.88	0.88	0.97	0.97	Not applicable
CDS	0.94	0.94	0.91	0.96	0.997
rRNA	0.91	0.91	0.97	0.91	0.995
tRNA	0.97	0.97	0.61	0.88	0.9996
total	0.90	0.90	0.89	0.95	0.87

Retained fractions are the averages of all replicates for each dataset.

^*a*^Before mapping, we removed a large NUMT on chromosome 2 of the *A. thaliana* nuclear genome.

^*b*^Before mapping, we removed the second copy of the large, inverted repeat (∼26 kb) in the *A. thaliana* ptDNA.

### XR-seq coverage of organellar vs nuclear genomes

We next compared the depth of XR-seq coverage (computed as RPKM) genome of the organellar and nuclear genomes (Table 2). In the *S. cerevisiae* and *A. thaliana* datasets, organellar XR-seq coverage was roughly one-third to two-fold that of the nuclear genome, while in the *D. melanogaster* data coverage of the mtDNA was over 50-fold that of the nuclear genome. Note that these estimates should not be directly interpreted as measures of the relative rates of degradation or repair in nuclear vs organellar DNA because they do not adjust for differences in organellar genome copy per nuclear genome, a parameter known to be highly variable under different life stages (Shen *et al.* 2019), tissue and cell types (Herbers *et al.* 2019; O’Hara *et al.* 2019), and physiological conditions (Göke *et al.* 2020). The relative rates of pyrimidine dimer formation in organellar vs nucDNA will also impact rates of repair, and estimates of the relative damage rates vary among species (Kalinowski *et al.* 1992; Ledoux *et al.* 1992; Takahashi *et al.* 2011) and depend on methods of detection (Gonzalez-Hunt *et al.* 2018).

**Table 2.** Organellar vs nuclear XR-seq coverage (as RPKM).

	*S. cerevisiae* mtDNA: CPD	*S. cerevisiae* mtDNA: (6-4)PP	*A. thaliana* mtDNA: CPD	*A. thaliana* ptDNA: CPD	*D. melanogaster* mtDNA: CPD
Organellar RPKM	25.0	72.1	11.7	13.6	424.9
Nuclear RPKM	82.7	82.3	8.4	8.4	6.9
Ratio: org/nuc RPKM	0.30	0.88	1.39	1.62	61.58

### Unique length distributions of organellar-mapping reads

**Fig. 2.**
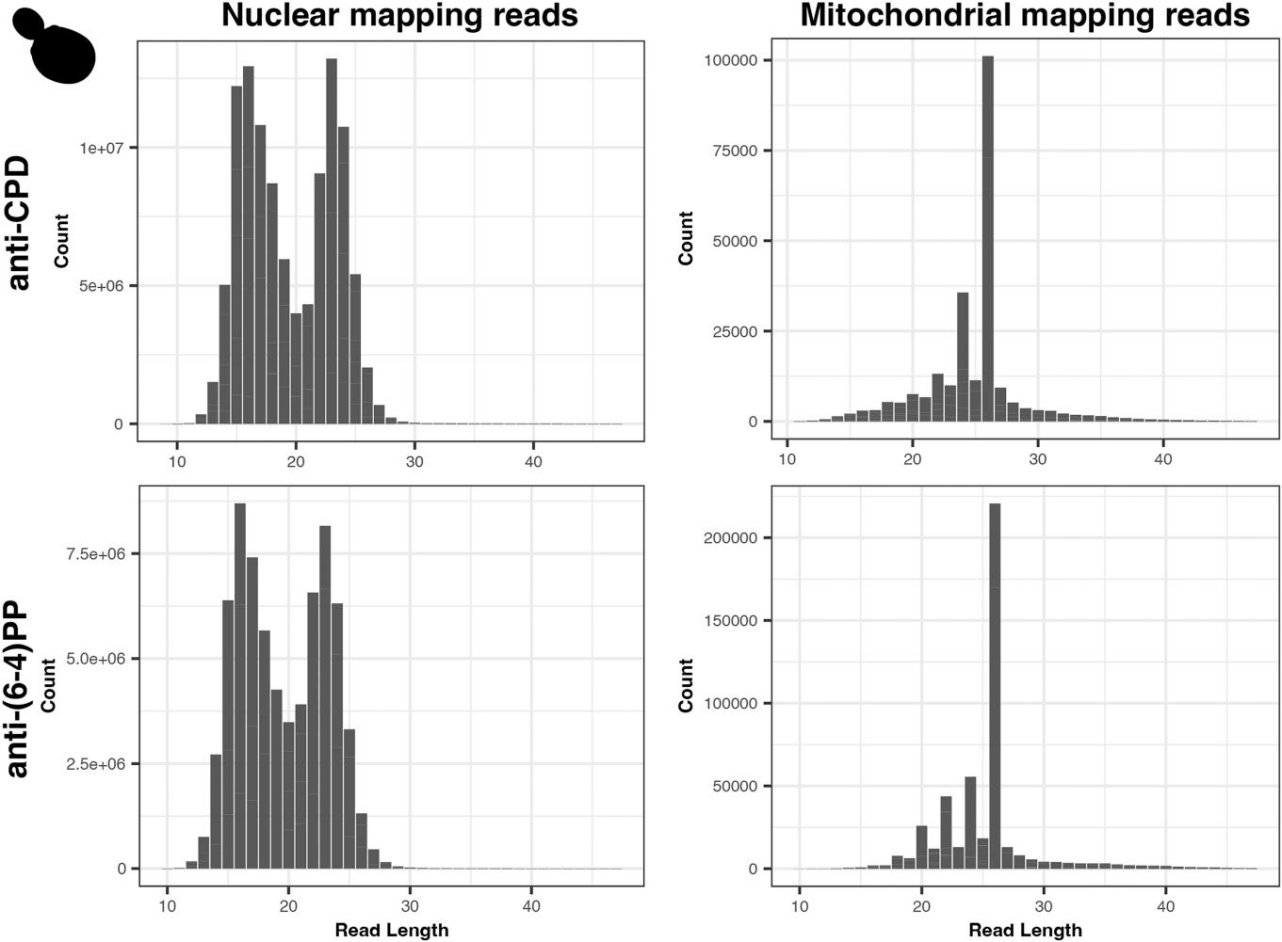
Read length distributions of nuclear and mitochondrial reads from anti-CPD and anti-(6-4)PP libraries from *S. cerevisiae.* These distributions exhibited a high degree of repeatability across samples and conditions. Pearson's correlation analyses reveal significant correlations between the anti-CPD and anti-(6-4)PP read length distributions (*R* = 0.9555, *P* = 1.6E-11) as well as between anti-CPD (5 vs 20 minutes; *R* = 0.9951, *P* = 2.2E-16) and anti-(6-4)PP (5 vs 20 minutes; *R* = 0.9765, *P* = 3.92E-14) timepoints.

**Fig. 3.**
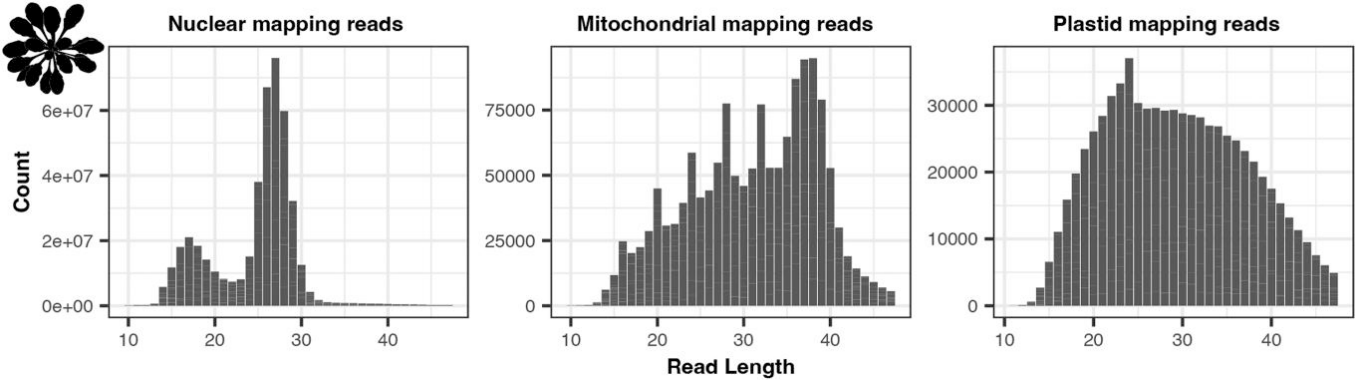
Read length distributions of nuclear, mitochondrial, and plastid mapping reads from the *A. thaliana* anti-CPD libraries. Pearson's correlation analyses reveal significant correlations between in the mtDNA read length distributions between time points (2 vs 5 hours; *R* = 0.9899, *P* = 2.2E-16).

We next analyzed the length of XR-seq fragments mapping to organellar and nuclear genomes. For all datasets, the organellar-mapping reads contain unique length distributions compared to those mapping to the nuclear genomes. As reported in the initial publication (Li *et al.* 2018), there are two peaks in the *S. cerevisiae* nucDNA mapping reads (in both anti-CPD and anti-(6-4)PP datasets), one derived from the primary excision products (23 nt) and the other (∼16 nt) presumably resulting from the 5′ degradation of the primary excision products (Fig. 2, Supplementary Figs. 1 and 6). The *S. cerevisiae* CPD and 6-4(PP) mtDNA-mapping reads show distinct peaks at read lengths of 26, 24, 22, and 20 nt (Fig. 2, Supplementary Figs. 2 and 7). The largest mtDNA peak of 26 nt is longer than the peak length in the nuclear-mapping reads of 23 nt. The *A. thaliana* mtDNA read length distributions also differ from the nucDNA read length distributions (Fig. 3, Supplementary Fig. 11). In the *A. thaliana* mtDNA read length distribution, there is a cluster of reads 36–39 nt in length, with additional distinct peaks in read lengths of 32, 28, 24, 20, and 16 nt (Fig. 3, Supplementary Fig. 12). Therefore, the patterns in these datasets were similar, but the peaks were spaced at different intervals (2 nt in *S. cerevisiae* and 4 nt in *A. thaliana*).

**Fig. 4.**
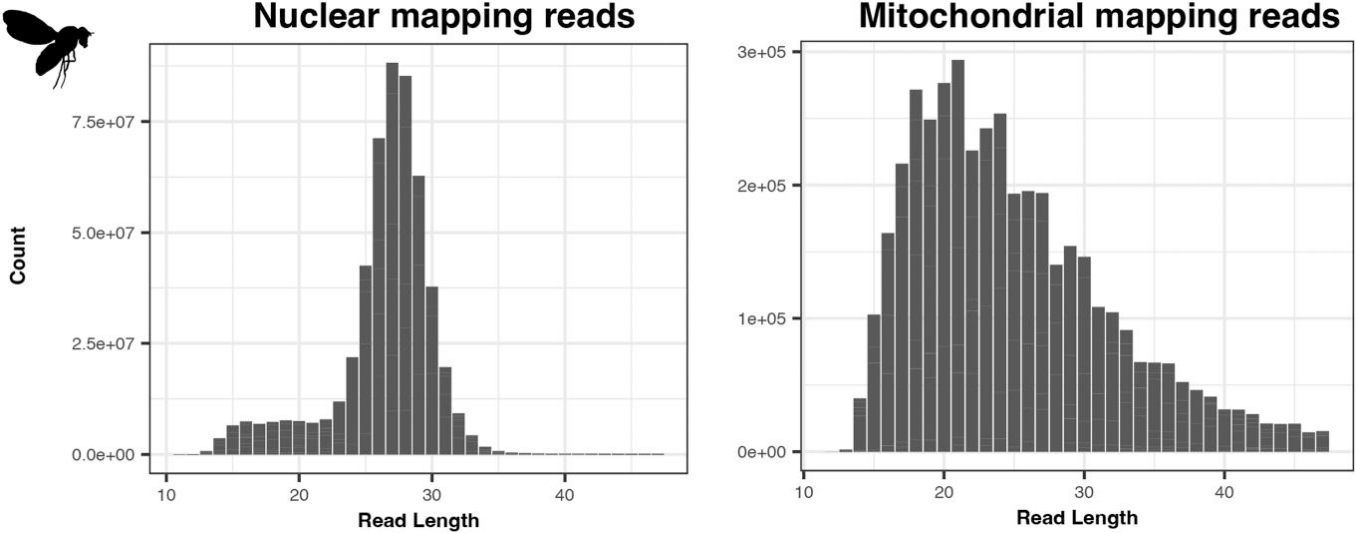
Read length distributions of nuclear and mitochondrial mapping reads from the *d. melanogaster* anti-CPD libraries.

The *A. thaliana* ptDNA read length distribution lacks distinct peaks occurring at regular intervals and instead contains a single, less extreme peak comprised of reads 24 nt in length (Fig. 3, Supplementary Fig. 13). The *D. melanogaster* mtDNA-mapping reads have a different read length distribution compared to the nuclear-mapping reads (Fig. 4, Supplementary Figs. 19 and 20), but the mtDNA-mapping reads lack the discrete peaks we observed in *S. cerevisiae* and *A. thaliana* organellar reads.

The origins of the distinct peaks in the XR-seq read length distributions of the *S. cerevisiae* and *A. thaliana* datasets are unclear. It is possible that these DNA fragments are derived from a mitochondrial-specific repair pathway for photodamage repair. Alternatively, the abundance of reads of certain lengths could arise from mtDNA degradation, which would represent a previously uncharacterized mechanism of damage-induced mtDNA degradation that results in fragments of specific lengths. One possibility is that the regular packaging of mitochondrial nucleoids could result in areas that are exposed to initial DNA damage or to subsequent incision/degradation, thereby affecting the length profile of mtDNA fragments after UV exposure. For example, the mtDNA-binding protein ABF2 has been shown to protect yeast mtDNA from oxidative damage (O’Rourke *et al.* 2002) and the mammalian homolog TFAM is known to bind mtDNA every ∼16 bps (Kukat *et al.* 2011).

XR-seq experiments in *E. coli* reveal somewhat similar patterns in the sense that there are a few read lengths that account for most of the reads in the length distribution, except that in *E. coli* most reads are of 10 or 13 nt in length (Adebali *et al.* 2017b). Interestingly, there is only a single peak of 13 nt in excision assays with *E. coli* mutants lacking *UvrD*, presumably because the primary 13mer oligonucleotide is unable to dissociate from the UvrB–UvrC heterodimer without the activity of the UvrD helicase and is therefore inaccessible to the exonucleases that degrade the oligonucleotide from the 3′ end (Adebali *et al.* 2017a). In the *S. cerevisiae* nucDNA-derived reads, TT peaks consistently occur 6 nt from the 3′ ends of reads, including in reads less than 23 nt (the length of the primary excision product in nucDNA NER), suggesting that nucDNA-derived reads are degraded from the 5′ ends. Given that secondary excision products have been shown to arise through exonuclease degradation of a primary oligonucleotide in *E. coli* and in the *S. cerevisiae* nucDNA NER, we hypothesize that the 26-nt peak in the *S. cerevisiae* mtDNA may be a “primary” product, with the less abundant 24-, 22-, and 20-nt oligonucleotides arising through degradation.

In the *S. cerevisiae* nuclear genome, photodamage results in the simultaneous activation of NER and HR, since transcriptional or replication fork stalling at damaged sites can result in DNA nicks, single-stranded gaps, or DSBs, all of which can stimulate HR (Toussaint *et al.* 2010). The importance of the cross-talk between NER and HR is exemplified by increased UV sensitivity in yeast lacking HR machinery (Gangavarapu *et al.* 2007). Interestingly, many genes involved in yeast nuclear HR are also important for repair of DSBs in the yeast mtDNA (Stein *et al.* 2015). Plants also rely heavily on HR for repair of DSBs and single-stranded nicks in mtDNA (Gualberto and Newton 2017). It is, therefore, possible that the discrete peaks in the *A. thaliana* and *S. cerevisiae* read length distributions may result from a recombination-based response to DSBs that arises as a downstream consequence of photodamage.

Attempts to visualize and validate the read length distributions observed in XR-seq data with a conventional excision assay found that the *S. cerevisiae* mtDNA signal was undetectable above background, even when the nucDNA signal was reduced by using a nuclear NER-deficient mutant strain background (*rad14Δ*) (Supplementary Fig. 24). It is likely that the signal of repair or degradation from the mtDNA is relatively weak compared to the noise of the assay (see faint gray smear in every lane of Supplementary Fig. 24). Future efforts to identify mtDNA fragments in excision assays may benefit from increased sample volumes and from physically isolating mitochondria from the cell suspensions before immunoprecipitation with antidamage antibodies.

### Preferential positioning of pyrimidines within organellar-mapping XR-seq reads

**Fig. 5.**
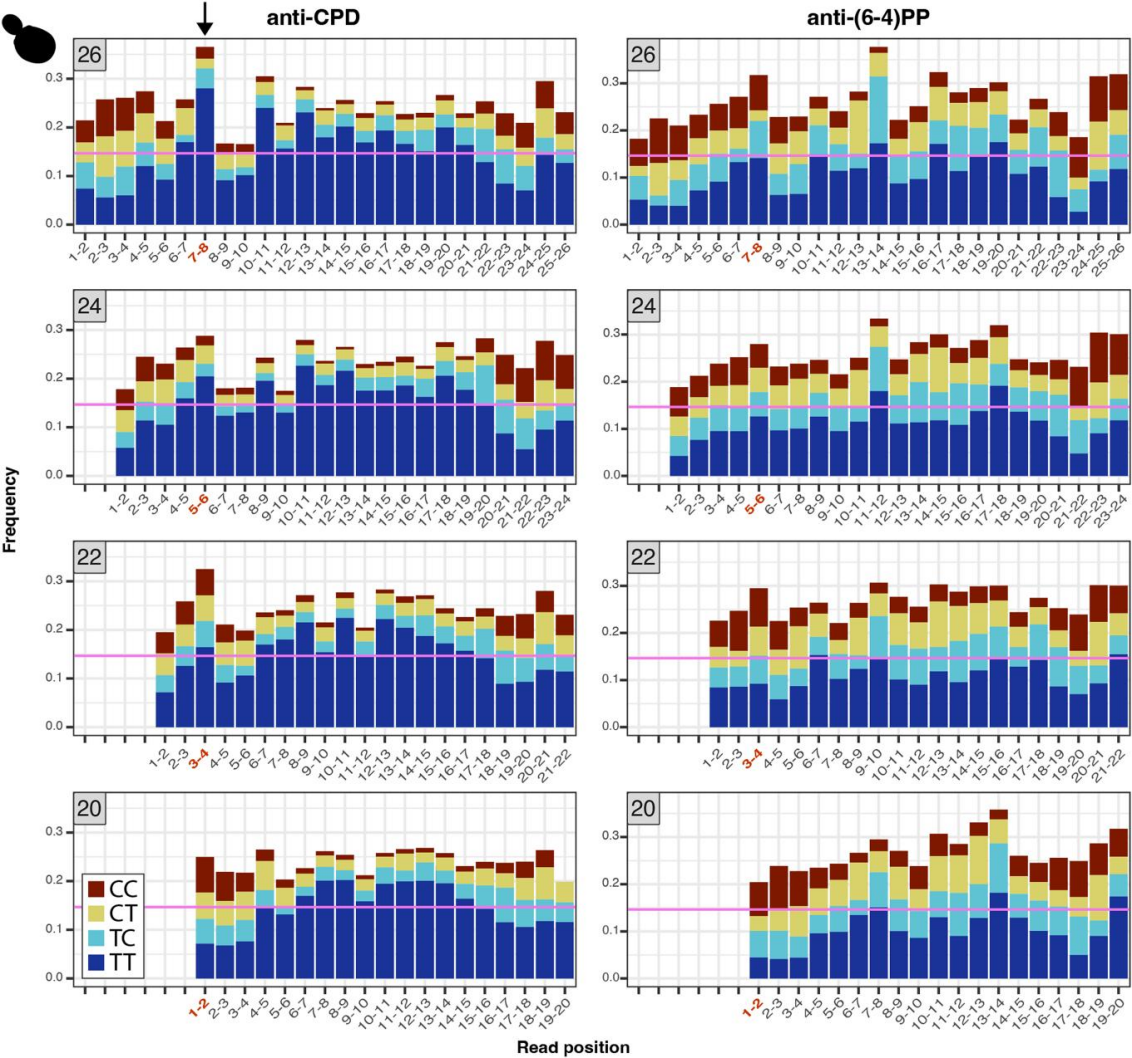
Di-pyrimidine frequencies in the most abundant read length classes (26-, 24-, 22-, and 20-nt) from the *S. cerevisiae* anti-CPD libraries. Read lengths are denoted in the gray boxes at the top left of each panel. The pink horizontal lines show the frequency of TT dinucleotides in the *S. cerevisiae* mtDNA, providing a null expectation for TT dinucleotide frequencies in the XR-seq reads. Positions with TT peaks in the 26-nt reads are in red, and the equivalent positions in the 3′ aligned (right aligned) 24-, 22-, and 20-nt reads are also in red. We approximated the 95% confidence interval as two times the standard error of the expected TT frequency given the number of reads included for each di-pyrimidine calculation. Given the large number of reads analyzed, 95% confidence intervals are very small, ranging from 0.1472 ± 0.0022 for the CPD 26-nt reads to 0.1472 ± 0.0081 for the CPD 20-nt reads. As a result, all blue bars that appear above the pink line in the figure represent a significant statistical enrichment relative to the expectation and its 95% confidence interval.

**Fig. 6.**
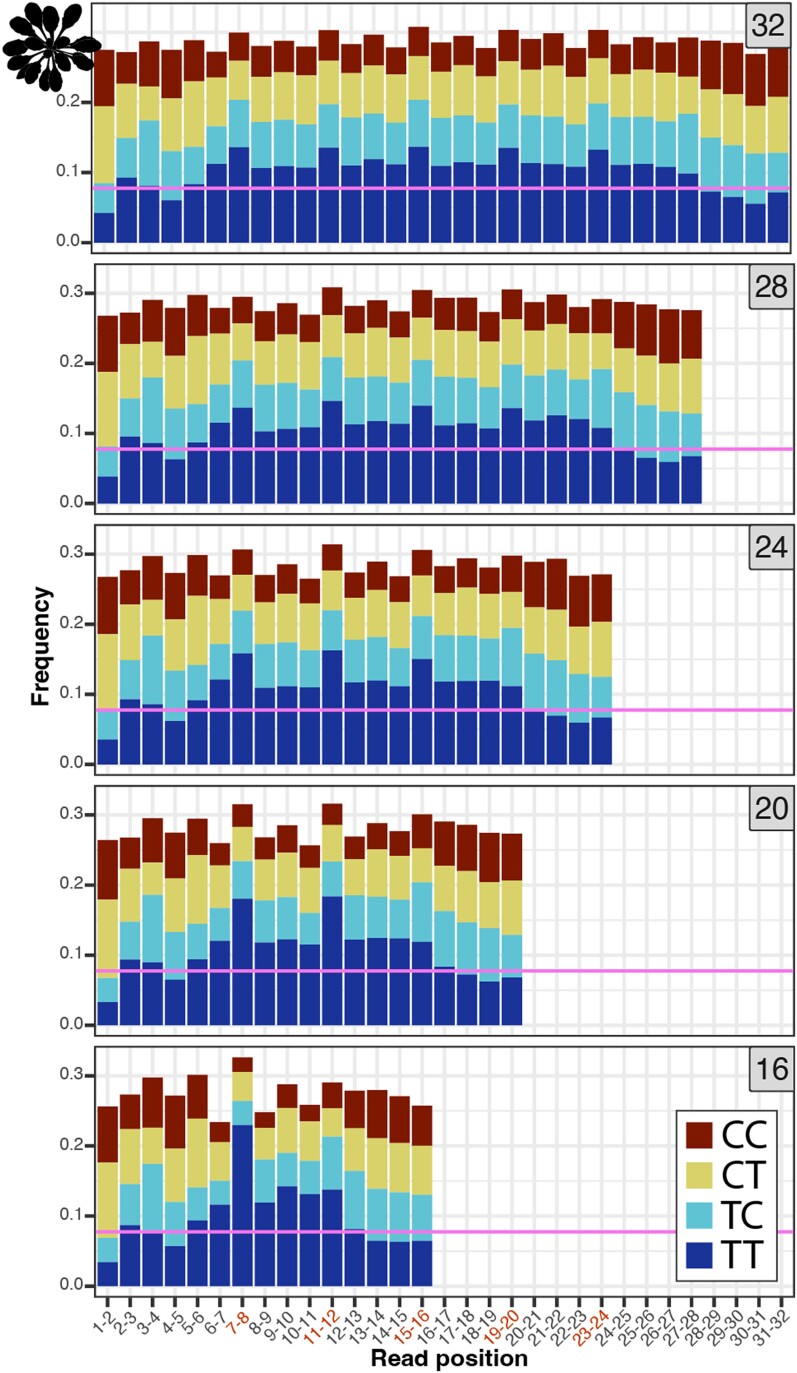
Di-pyrimidine frequencies in the most abundant read length classes (32, 28, 24, 20, and 16 nt) from the *A. thaliana* mtDNA-mapping reads. Read lengths are denoted in the gray boxes at the top right of each panel. The pink horizontal lines show the frequency of TT dinucleotides in the *A. thaliana* mtDNA, providing a null expectation for TT dinucleotide frequencies in the XR-seq reads. See Fig. 5 for a description of calculating 95% confidence intervals around this expectation. These confidence intervals were very small due to the large number of reads, ranging from 0.0743 ± 0.0033 for the 16-nt reads to 0.0743 ± 0.0018 for the 32-nt reads.

We analyzed the nucleotide and di-pyrimidine frequencies of all the organellar-mapping reads, focusing especially on the dominant read lengths in the *S. cerevisiae* and *A. thaliana* mtDNA datasets (shown in Figs. 2 and 3, respectively). In the 26-nt *S. cerevisiae* mtDNA-mapping reads (the most frequent length class) in the CPD dataset, adjacent thymines (TTs) are most abundant at positions 7–8, with additional smaller peaks spaced at 2-nt intervals, starting at position 10 (left panel of Fig. 5; di-pyrimidine frequencies of all the (6-4)PP-mapping reads including rare size classes are shown in Supplementary Fig. 3). The 24-nt reads show a similar TT peak pattern, though it is shifted forward two positions compared to the pattern in the 26-nt reads (i.e. a peak at positions 5–6, followed by secondary peaks starting at positions 8, 10, and 12). In the 22-nt reads, the TT peaks are shifted forward four positions. Therefore, the TT peaks fall in the same position when the 26-, 24-, and 22-nt reads are 3′ or right aligned as they are in Fig. 5. In the (6-4)PP dataset, the TT peaks also fall in similar positions when the most common reads (26-, 24-, 22-, and 20-nt) are 3′ aligned (right panel of Fig. 5, di-pyrimidine frequencies of all the (6-4)PP mapping reads including rare size classes are shown in Supplementary Fig. 8), though the TT peak at positions 7–8 does not rise above the null expectation derived from the frequency of TTs in the *S. cerevisiae* mtDNA. As in the *S. cerevisiae* dataset, the TT peaks in the *A. thaliana* mtDNA mapping reads fell in the same position when the most frequent read lengths are aligned (in this case reads 32, 28, 24, 20 and 16 nt long). For the *A. thaliana* dataset, this pattern holds regardless of whether the reads are left (5′) aligned (as in Fig. 6; di-pyrimidine frequencies of all the mtDNA-mapping reads including rare size classes are shown in Supplementary Fig. 14) or right (3′) aligned (not shown).

**Fig. 7.**
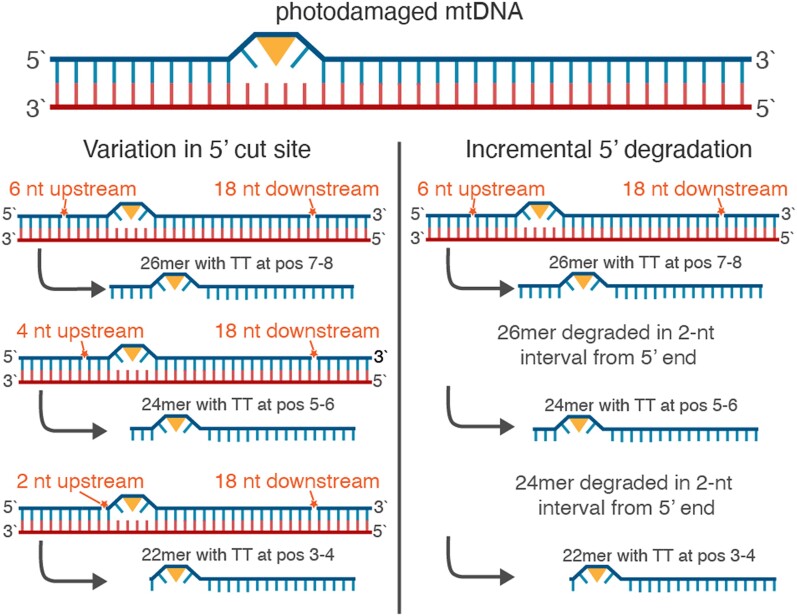
Proposed alternative explanations for unique read length distributions and dinucleotide composition patterns in the *S. cerevisiae* anti-CPD and anti (6-4)PP datasets.

The observed patterns in the mtDNA-derived XR-seq reads may arise through mtDNA degradation or through an incision-based repair process. In either scenario, we propose a potential mechanism in which “primary” 26-nt DNA fragments may be degraded in 2-nt intervals from the 5′ end to produce 24-, 22-, and 20-nt products (Fig. 7, right panel). Alternatively, incisions of 6-, 4-, or 2-nt upstream of a CPD could yield the 26-, 24-, and 22-nt products, respectively (Fig. 7, left panel). Under the model that these DNA fragments arise through wholesale mtDNA degradation, rather than a specific incision-based pathway, we hypothesize that TT dimers inhibit mtDNA-degrading nucleases from accessing upstream or downstream nucleotides, resulting in the enrichment of di-pyrimidines at internal locations in the DNA fragments, though we are not aware of examples of exonucleases stalling at such distances from pyrimidine dimers in the literature. Instead, previous efforts to understand the fate of excised oligonucleotides generated during NER in nucDNA have identified multiple exonucleases that can remove nucleotides up to a dimer (Kemp and Sancar 2012; Hu *et al.* 2013; Adebali *et al.* 2017a; Kim *et al.* 2022). Similarly, if the DNA fragments in the *A. thaliana* dataset (Fig. 6) are arising through targeted incisions, we posit that these incisions occur primarily either 6-, 10-, 14-, 18-, or 22-nt upstream of a CPD and either 8-, 12-, 16-, 20-, or 24-nt downstream of a CPD. Alternatively, the 4-nt spacing of pyrimidine dimers could be explained by regular degradation of a primary excision product of undetermined length. Yet another possibility might be that if these fragments are arising through mtDNA degradation, it would again appear that degrading nucleases are unable to access DNA within a certain distance of pyrimidine dimers.

DNA fragments with dimers on the end are difficult to study because they can be recalcitrant to elongation by terminal transferase enzymes necessary for radiolabeling and likely to ligation of adaptors necessary for XR-seq (Kim *et al.* 2022). Therefore, it is possible that “dimer-capped” mtDNA molecules generated through exonuclease activity up to the dimer would be undetectable in XR-seq datasets. Previous XR-seq studies have also suggested that adaptor ligation biases may drive variation in nucleotide composition within reads (Li *et al.* 2017), especially at read ends where adaptors are ligated. We cannot rule out the possibility that adaptor ligation biases are responsible for the enrichment of TT dinucleotides at specific positions (e.g. positions 7–8 in 26-nt *S. cerevisiae* CPD reads). It is also possible that the antidamage antibodies favor binding to certain sequence motifs, which could lead to the preferential positioning of pyrimidine peaks within reads. However, we view ligation or antibody biases as unlikely explanations given that the enrichment patterns differ greatly across species (e.g. *S. cerevisiae* vs *D. melanogaster*) and across genomes of the same species (e.g. *S. cerevisiae* nucDNA vs mtDNA or *A. thaliana* ptDNA vs mtDNA) despite use of the same antibodies and adaptors. Ligation biases seem especially unlikely given that the TT enrichment is internal to the fragments and not directly at the ligated ends and often outside the random 5-nt sequence used for adaptor annealing. Further, the enriched positions shift relative to the 5′ end depending on the length of read (Figs. 5, 7), whereas if ligation biases were responsible, we would expect a consistent position of pyrimidines relative to the ends of reads.

The *A. thaliana* ptDNA and *D. melanogaster* mtDNA reads lack obvious di-pyrimidine patterns (Supplementary Figs. 15 and 21). Interestingly, the *D. melanogaster* mtDNA-mapping reads show extreme nucleotide biases at both the 5′ and 3′ ends of reads (Supplementary Fig. 22). Such biases may be driven by biased composition of the overhanging Ns that allow for adaptor annealing. However, nuclear-mapping XR-seq reads do not display extreme nucleotide biases at read ends (see Fig. 1c; Deger *et al.* 2019). End biases are also mostly absent from the *S. cerevisiae* and *A. thaliana* organellar-mapping reads (Supplementary Figs. 4,9, 16, and 17, respectively), which were created with the same or similar adaptors. Another explanation could be that in the *D. melanogaster* mtDNA, distance from a CPD is not important in determining upstream or downstream incision sites, and instead, local sequence contexts drive incision locations. Such a phenomenon would also explain why the *D. melanogaster* read length distribution lacks discrete peaks (Supplementary Fig. 20) and why the *D. melanogaster* mtDNA-mapping reads lack an enriched localization of di-pyrimidines (Supplementary Fig. 21).

### Variation in the distribution of XR-seq reads among genomic regions

We determined the location of the organellar-mapping reads as either intergenic, CDS (protein coding), intronic, tRNA coding, or rRNA coding. In both *S. cerevisiae* datasets (CPD and (6-4)PP), we find elevated coverage of genic regions (CDS, rRNA and tRNA) compared to coverage in intergenic regions (Supplementary Figs. 5 and 10, left panel). This pattern is consistent with trends in the *S. cerevisiae* nuclear genome (Li *et al.* 2018), where increased genic XR-seq coverage is attributed to transcription-coupled NER (TC-NER). The increased coverage of the genic regions in these datasets is not driven by an imbalance in the relative amount of di-thymines between regions, as the yeast mtDNA intergenic sequences have elevated di-thymines compared to genic sequences (Supplementary Table 2). Another feature of TC-NER is increased coverage of the template DNA strand compared to the coding DNA strand. After controlling for differences in the numbers of di-pyrimidines on the template DNA strand compared to the coding DNA strand, we find elevated coverage of the template strand compared to the coding strand in the tRNA coding regions of the genome in both *S. cerevisiae* datasets (CPD and (6-4)PP; Supplementary Figs. 5 and 10, right panel). However, CDS, intronic, and rRNA regions show no difference in coverage of the template vs coding strand or slightly elevated coverage of the coding strand compared to the template strand, which is inconsistent with expectations of TC-NER.

In the *A. thaliana* mtDNA, we see slightly elevated XR-seq coverage of the CDS compared to the intergenic regions of the genome, but rRNA and tRNA genes, which are typically expressed more highly than CDS regions (Belozerova *et al.* 2011; Pérez Di Giorgio *et al.* 2019 ), have XR-seq coverage below or near the level of intergenic sequence (Supplementary Fig. 18, top left panel). This suggests that increases in expression may not correlate with increased levels of incisions or repair activity as is observed in the *A. thaliana* nucDNA due to TC-NER (Oztas *et al.* 2018). In the *A. thaliana* ptDNA, we see relatively even levels of CDS and intergenic coverage but decreased coverage of rRNAs and tRNAs (Supplementary Fig. 18, top right panel), again opposite of the expectations under a TC repair model where more highly expressed genes receive increased NER protection. If the organellar-derived DNA fragments arise through organellar genome degradation rather than by uncharacterized repair pathways, variation in XR-seq read depth across genomic compartments may provide a snapshot of variation in damage formation. Supporting this idea, and in contrast to the *S. cerevisiae* results, the *A. thaliana* regions with the lowest abundance of di-thymines (specifically the tRNA and rRNA genes; Supplementary Table 2) also have the lowest relative XR-seq coverage (Supplementary Fig. 18). Therefore, the variation in coverage between regions in the *A. thaliana* organellar genomes appears to be correlated with the relative abundance of di-thymines between the regions (Supplementary Table 2). There are no large effect asymmetries in coding vs template strand in the *A. thaliana* data (Supplementary Fig. 18, bottom panels) except for in mtDNA and ptDNA rRNA genes, especially in the ptDNA where template coverage is roughly 2-fold that of the coding strand. It is difficult to know whether these asymmetries arise through variation in damage formation, uncharacterized repair pathways, or asymmetrical DNA degradation. Therefore, overall, we find very little support for the possibility that the observed DNA fragments produced in response to UV damage are dependent on transcriptional activity.

Differences in XR-seq coverage between genomic regions may also arise from different levels of pyrimidine dimer formation, which has been shown to vary across nucDNAs due to variation in local sequence motifs and nucleosome density (Mao *et al.* 2016). Organellar DNA lacks nucleosomes and is instead packaged in nucleoids, which can vary in protein components based on developmental and physiological status of a given organelle, but are generally assumed to confer many of the same protective benefits as nucleosomes (Bogenhagen 2012; Sakamoto and Takami 2018; Zhao 2019).

In the *D. melanogaster* mtDNA, we find a drastic reduction in coverage of the intergenic portion of the genome compared to the CDS, rRNA, and tRNA genes (Supplementary Fig. 23, top panel). Metazoan mtDNAs are extremely gene dense, so essentially all of the “intergenic” sequence in the *D. melanogaster* mtDNA is located in the AT-rich region of the genome, which serves as the mtDNA replication origin and termination sites. Given the preponderance of thymines in this region, one might expect an increase in CPD formation compared to other regions of the genome, making the lack of XR-seq read in this region intriguing (Supplementary Table 2). However, AT-rich sequences also experience negative amplification biases during the PCR stages of library construction (Aird *et al.* 2011; Wu *et al.* 2020a; Waneka *et al.* 2021), so comparisons of XR-seq coverage between regions of varied AT content must be made cautiously. Coverage is lower in on template strand than the coding strand in all genomic regions in the *D. melanogaster* mtDNA, opposite of expectations given TC-NER (Supplementary Fig. 23, bottom panel).

## Conclusion

Early studies that found no repair of UV-damaged mtDNAs in human and yeast cells (Clayton *et al.* 1974; Prakash 1975) helped shape the notion that mitochondria lack DNA repair altogether and that damaged mtDNA molecules are simply degraded, with undamaged copies serving as templates for new mtDNA synthesis (Druzhyna *et al.* 2008). While subsequent investigations have unveiled that specific types of mtDNA base damage such as deamination, simple alkylation, and oxidation can indeed be effectively repaired within the mitochondria, it is still generally accepted that all eukaryotes lack any pathway for repair of bulky DNA damage in mtDNAs (Kazak *et al.* 2012; Stein and Sia 2017; Alencar *et al.* 2019; Chevigny *et al.* 2020). MtDNA damage has been demonstrated to lead to mtDNA degradation in a variety of instances (Bess *et al.* 2012, 2013; Shokolenko *et al.* 2016; Moretton *et al.* 2017; Wang *et al.* 2017; Dan *et al.* 2020), but this process remains enigmatic, with open questions as to how damaged mtDNAs are distinguished from healthy mtDNAs, how damaged mtDNAs promote fusion and/or mitophagy (Bess *et al.* 2013; Wang *et al.* 2017; Doblado *et al.* 2021), and which enzymes actually degrade the mtDNA (Moretton *et al.* 2017; Matic *et al.* 2018; Peeva *et al.* 2018).

Our analysis of XR-seq experiments shows that mitochondrially derived DNA fragments of characteristic length and nucleotide composition are produced following mtDNA photodamage in both *S. cerevisiae* and *A. thaliana*. As we have laid out, we envision two potential mechanisms that could be responsible for productions of these DNA fragments: (1) an uncharacterized repair pathway functioning in mitochondria or (2) a previously uncharacterized programmed degradation of damaged mtDNA. Either of these possibilities point to the exciting prospect of novel maintenance or processing in response to exogenous damage.

A key next step in differentiating between these and other possible models will be to identify the specific molecular machinery that produces the observed DNA fragments in response to UV damage. Given the ancient bacterial ancestry of the mitochondria and the presence of other noncanonical, taxon-specific mtDNA repair machinery, it is difficult to make predictions about the evolutionary origins of the genes which may be involved in mtDNA photodamage response. In eukaryotic NER, the Cockanye syndrome (CS) genes *CSA* and *CSB* couple NER to transcription via the recognition of stalled RNA polymerases (Van Gool *et al.* 1997; Selby *et al.* 2023). Interestingly, in mammalian cells, *CSA* and *CSB* are also targeted to the mitochondria where they are thought to integrate with BER machinery to facilitate the elimination of oxidized purines in mtDNA (Kamenisch *et al.* 2010; Chatgilialoglu *et al.* 2022) likely through the recognition of stalled RNA polymerases (Scheibye-Knudsen *et al.* 2016). Meanwhile in bacteria, the coupling of NER to transcription is performed by the transcription repair coupling factor (*TRCF*) which recognizes stalled RNA polymerases and recruits the *UvrABC* machinery to excise the damage-containing oligomer (Deaconescu *et al.* 2007). Although all eukaryotes lack *UvrABC* genes, plants possess a *TRCF* homolog, which is dual-targeted to the mitochondria and the chloroplast and has an unknown molecular function (Gualberto and Newton 2017). The CS and *TRCF* genes may be good candidates for future work investigating the response to mitochondrial photodamage. Given that these genes are responsible for damage recognition, they may be involved whether the response to photodamage is mtDNA degradation or bulky DNA damage removal through a noncanonical repair pathway. XR-seq experiments may prove valuable for detecting perturbations in the lengths and internal di-pyrimidine positioning of mtDNA-derived DNA fragments in mutant lines lacking these damage recognition genes.

## Supplementary Material

iyae070_Supplementary_Data

## Data Availability

The scripts and unix commands used to analyze the publicly available XR-seq datasets are available via https://github.com/dbsloan/mtDNA_UV_damage. Supplementary material available at GENETICS online.
