# Supplementary material for: UV damage induces production of mitochondrial DNA fragments with specific length profiles
Source: Genetics. 2024 May 9;227(3):iyae070. doi: 10.1093/genetics/iyae070 (PMC11228841; doi:10.1093/genetics/iyae070)
Supplement: iyae070_Supplementary_Data [file iyae070_supplementary_data.zip › Supplemental_Figures_GENETICS-2024-307036.pdf]

SUPPLEMENTAL FIGURES

S. cerevisiae anti-CPD libraries

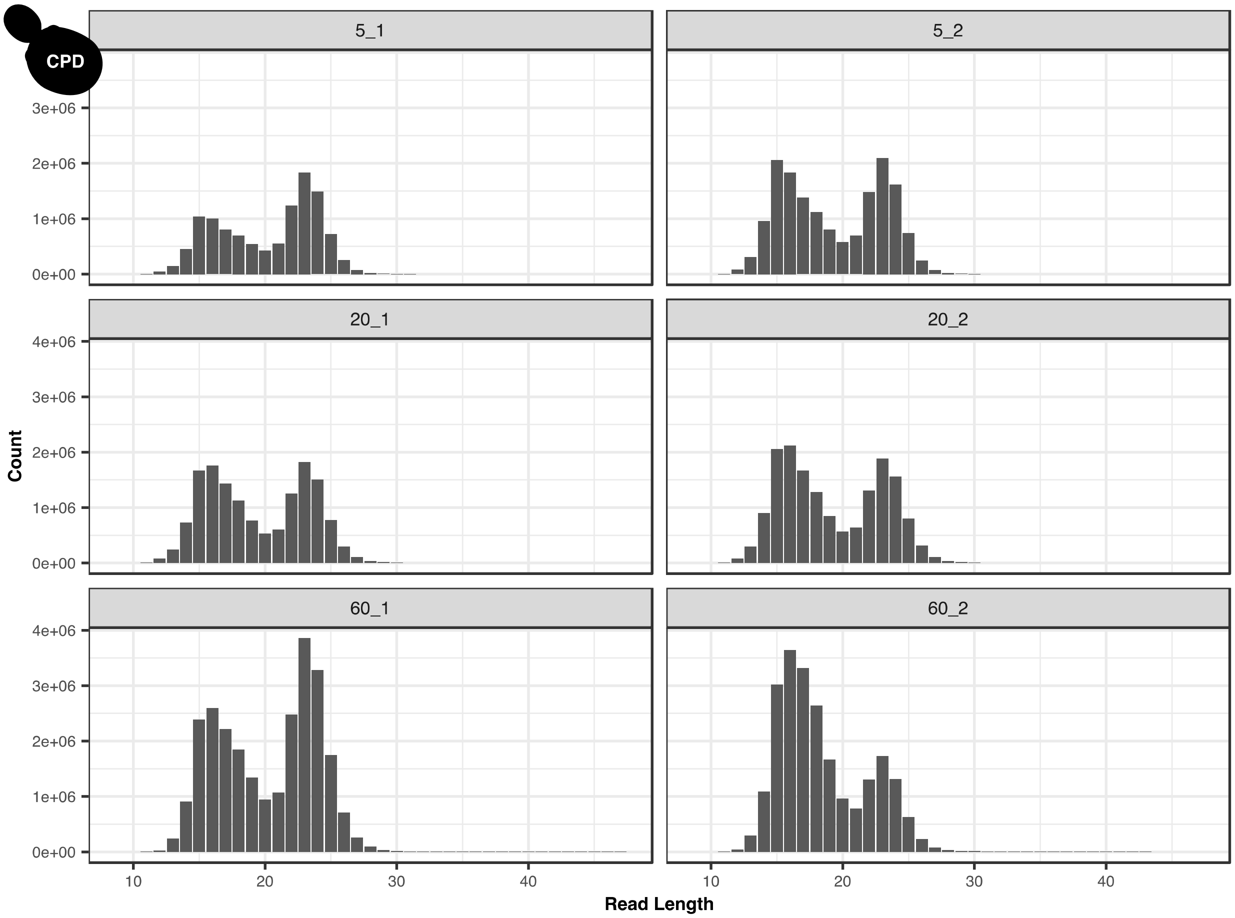

Figure S1. Length distributions of the nuclear-mapping reads from the *S. cerevisiae* anti-CPD libraries. The panel labels show the library ID, with the first number indicating the repair time (5, 20 or 60 minutes) and the second number indicating the replicate number (1 or 2).

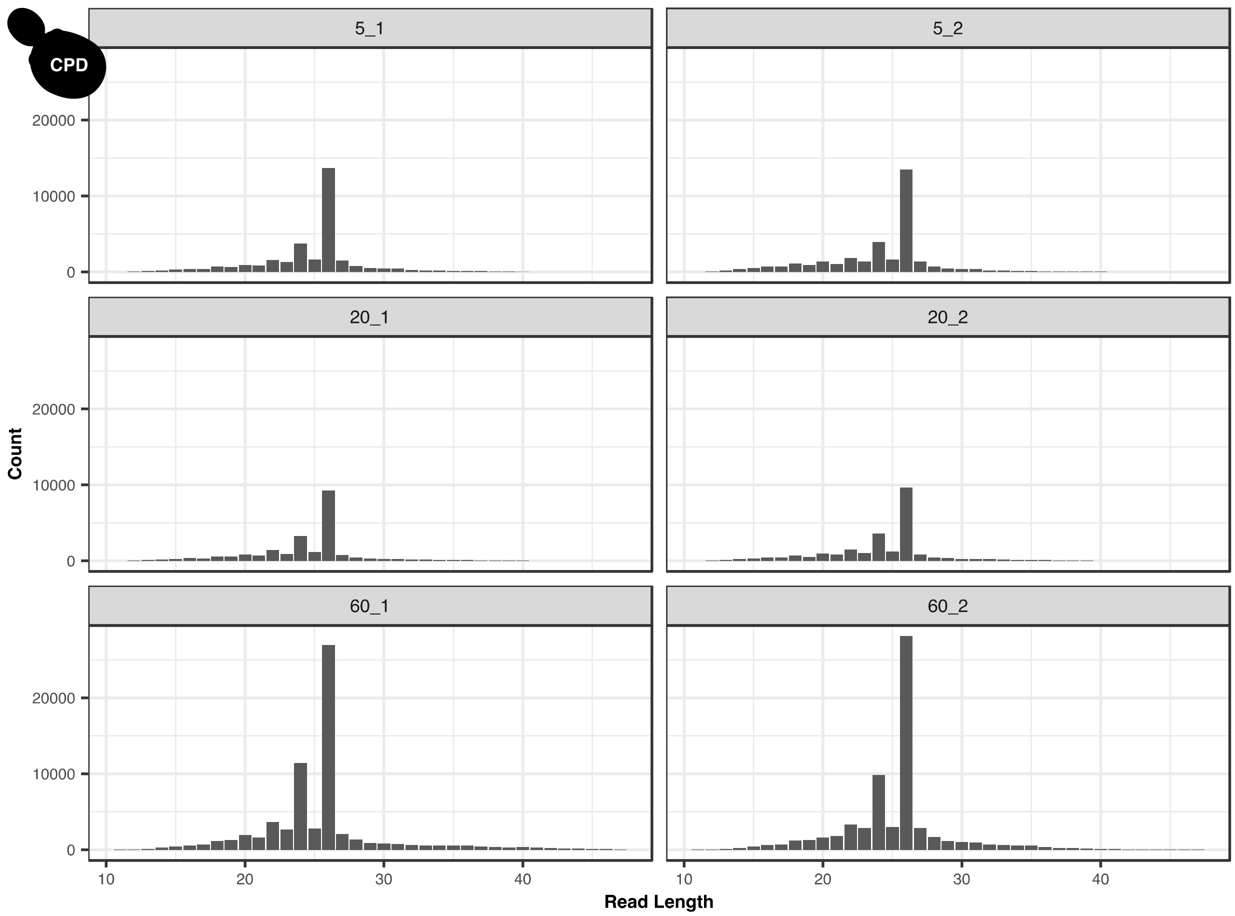

Figure S2. Length distributions of the mtDNA mapping reads from the *S. cerevisiae* anti-CPD libraries. The panel labels show the library ID, with the first number indicating the repair time (5, 20 or 60 minutes) and the second number indicating the replicate number (1 or 2).

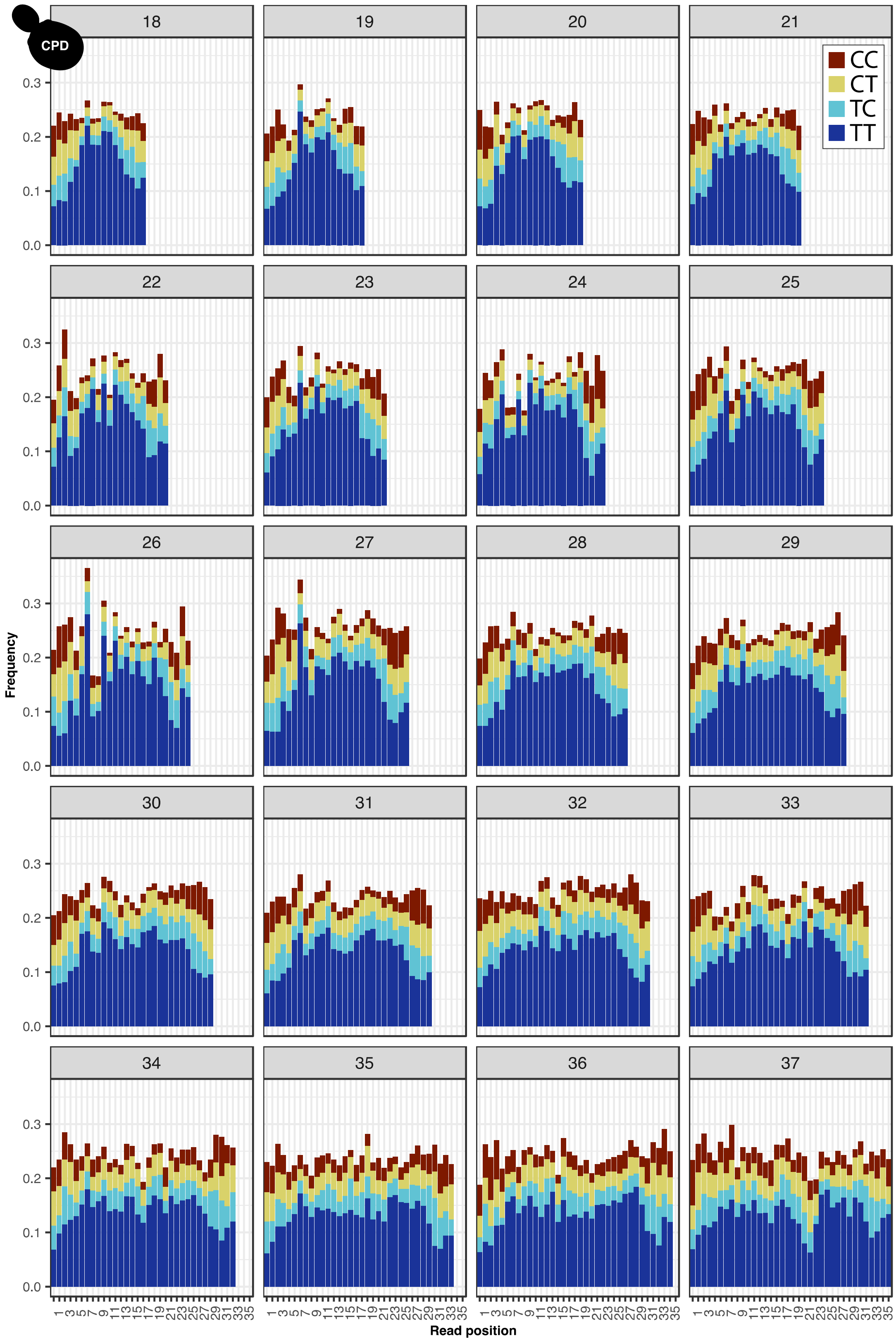

Figure S3. Di-pyrimidine frequencies in the mtDNA mapping reads from the *S. cerevisiae* anti-CPD libraries for all read-length classes.

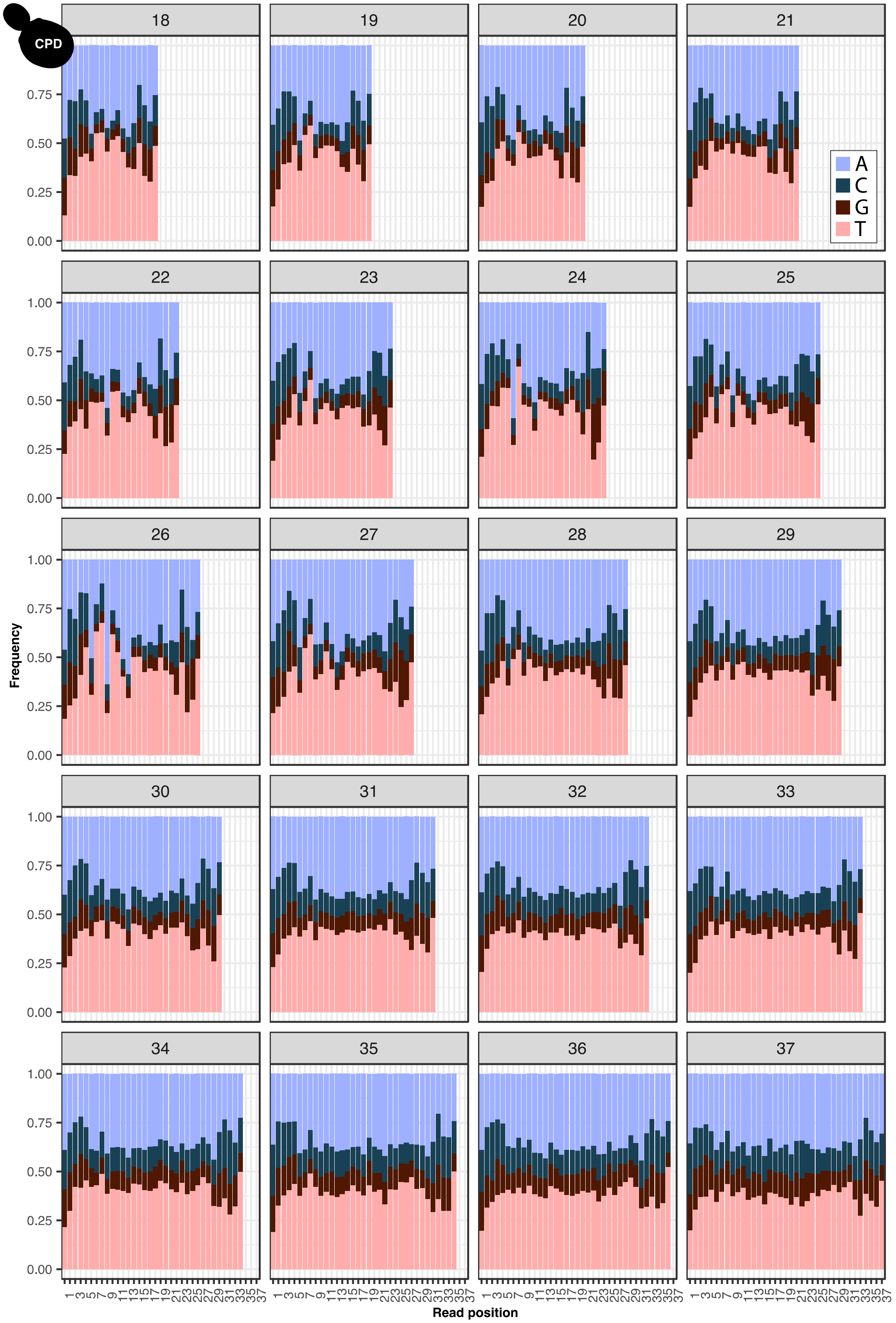

Figure S4. Nucleotide frequencies in the mtDNA mapping reads from the *S. cerevisiae* anti-CPD libraries for all read-length classes.

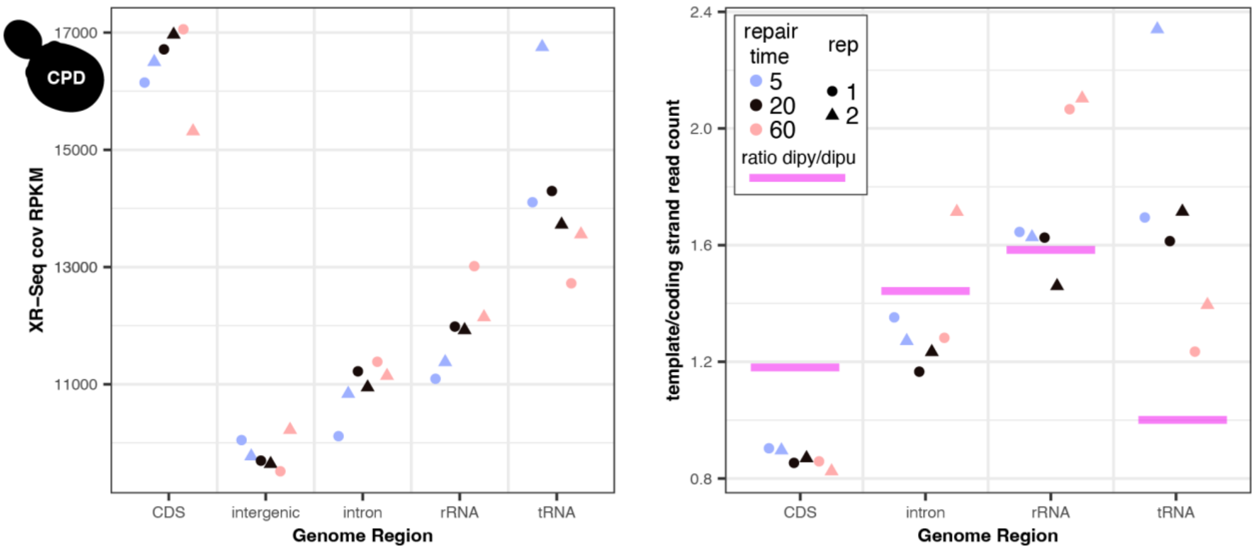

Figure S5. Depth of coverage (RPKM) and strand assymetry by genomic region in the mtDNA-mapping reads from the *S. cerevisiae* anti-CPD libraries. The pink bars show the ratio of dipyrimidines over dipurines on the coding strand, serving as an approximate null hypothesis for the ratio of XR-seq reads mapping to the template or coding strand.

***S. cerevisiae* anti-(6-4)PP libraries**

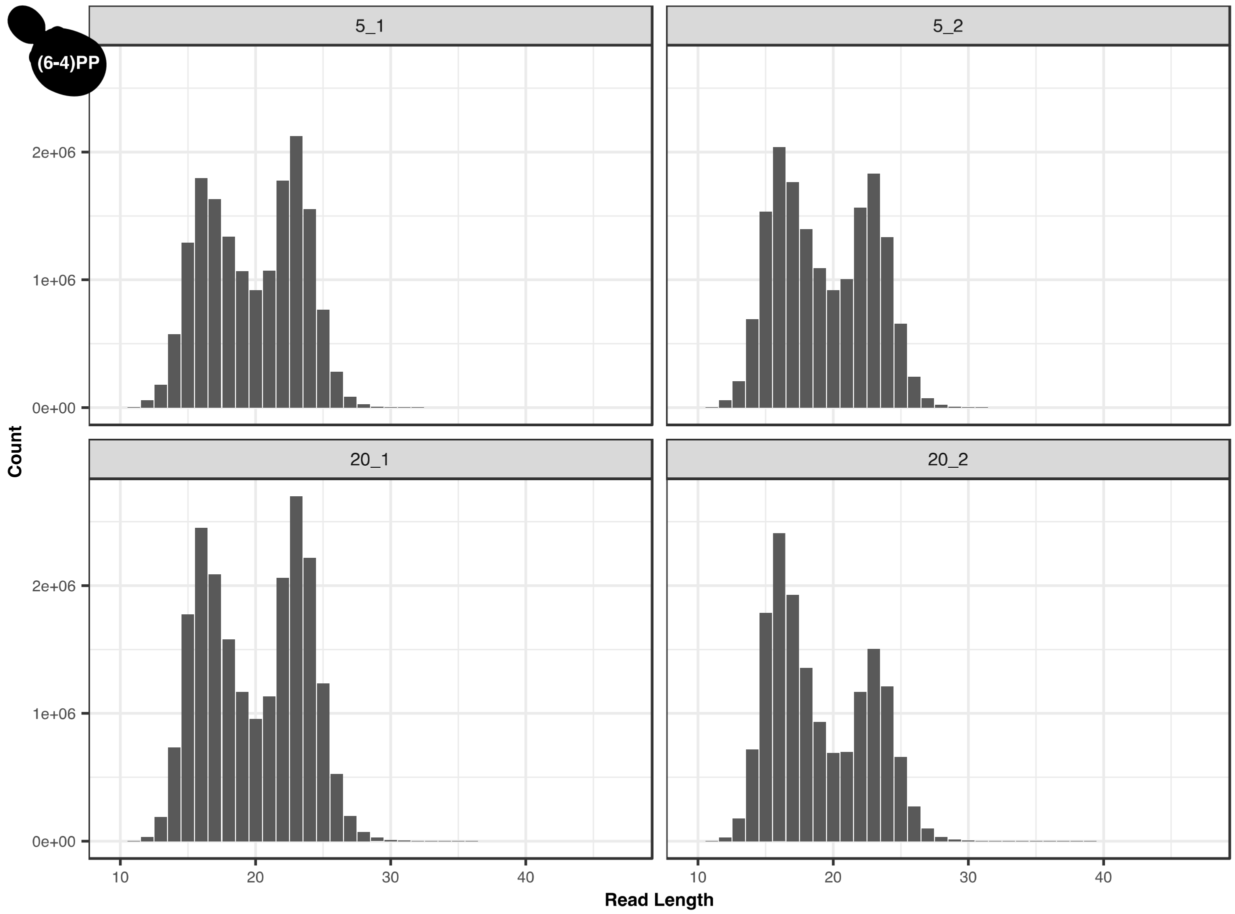

Figure S6. Length distributions of the nuclear-mapping reads from the *S. cerevisiae* anti-(6-4)PP libraries. The panel labels show the library ID, with the first number indicating the repair time (5 or 20 minutes) and the second number indicating the replicate number (1 or 2).

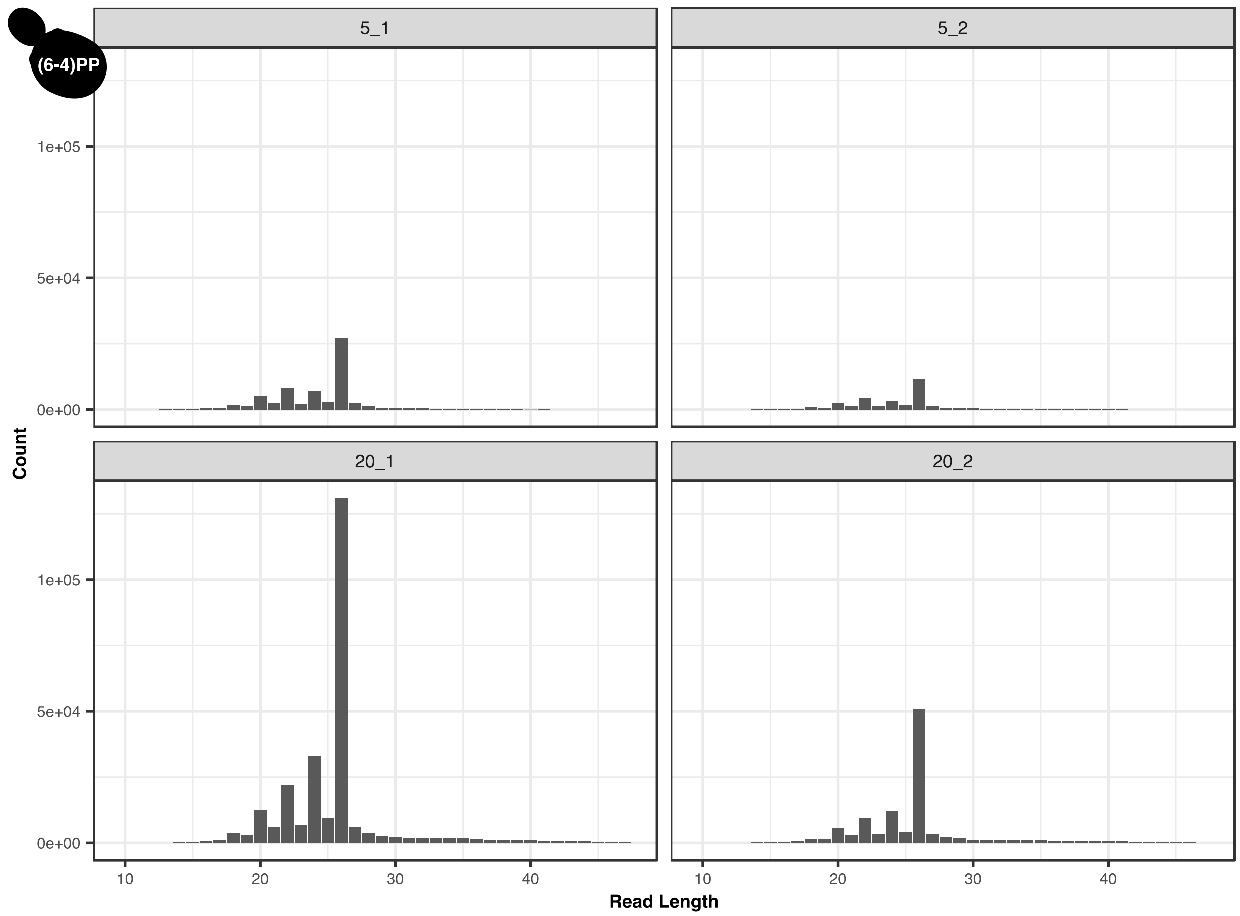

Figure S7. Length distributions of the mtDNA-mapping reads from the *S. cerevisiae* anti-(6-4)PP libraries. The panel labels show the library ID, with the first number indicating the repair time (5 or 20 minutes) and the second number indicating the replicate number (1 or 2).

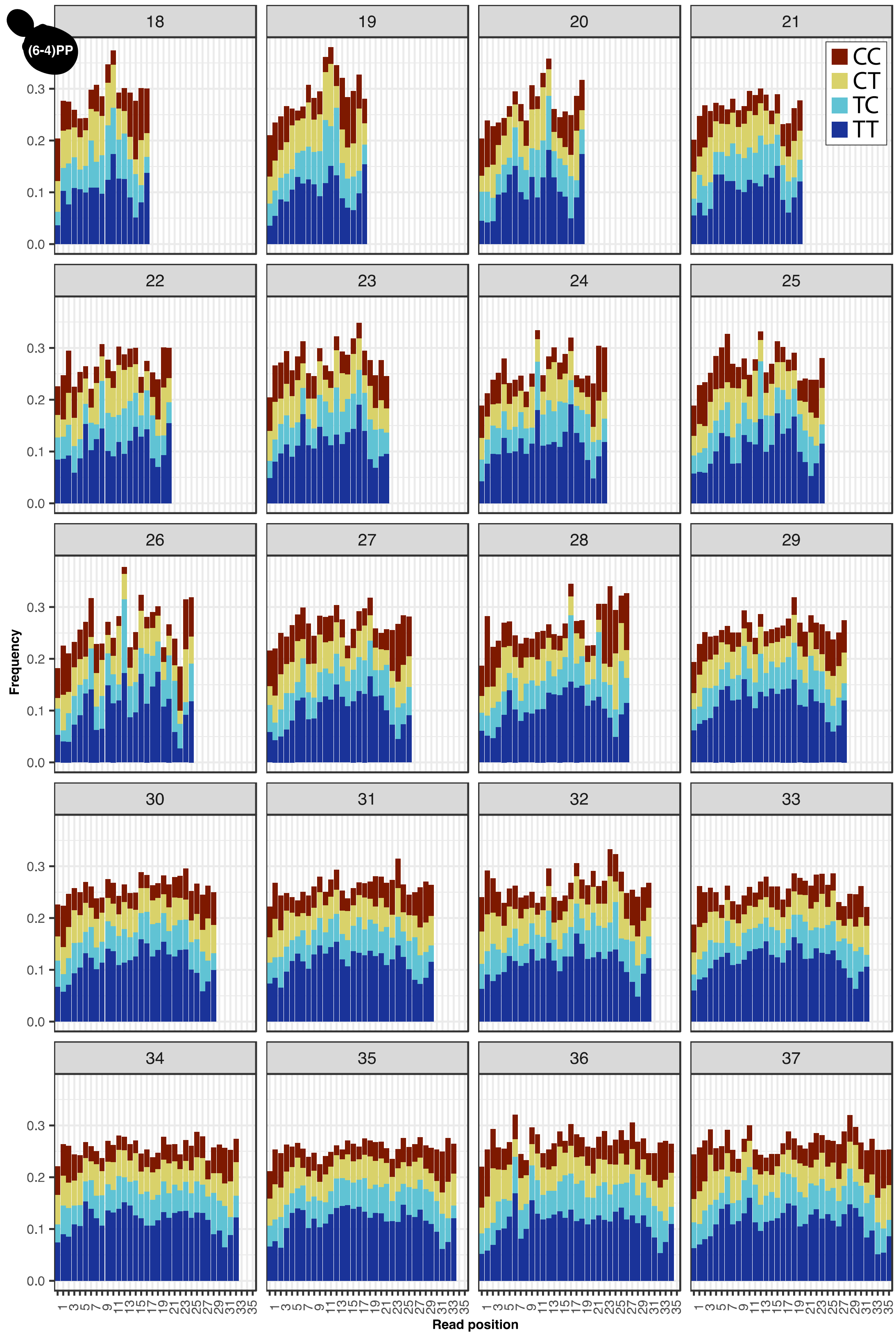

Figure S8. Di-pyrimidine frequencies in the mtDNA-mapping reads from the *S. cerevisiae* anti-(6-4)PP libraries for all read-length class.

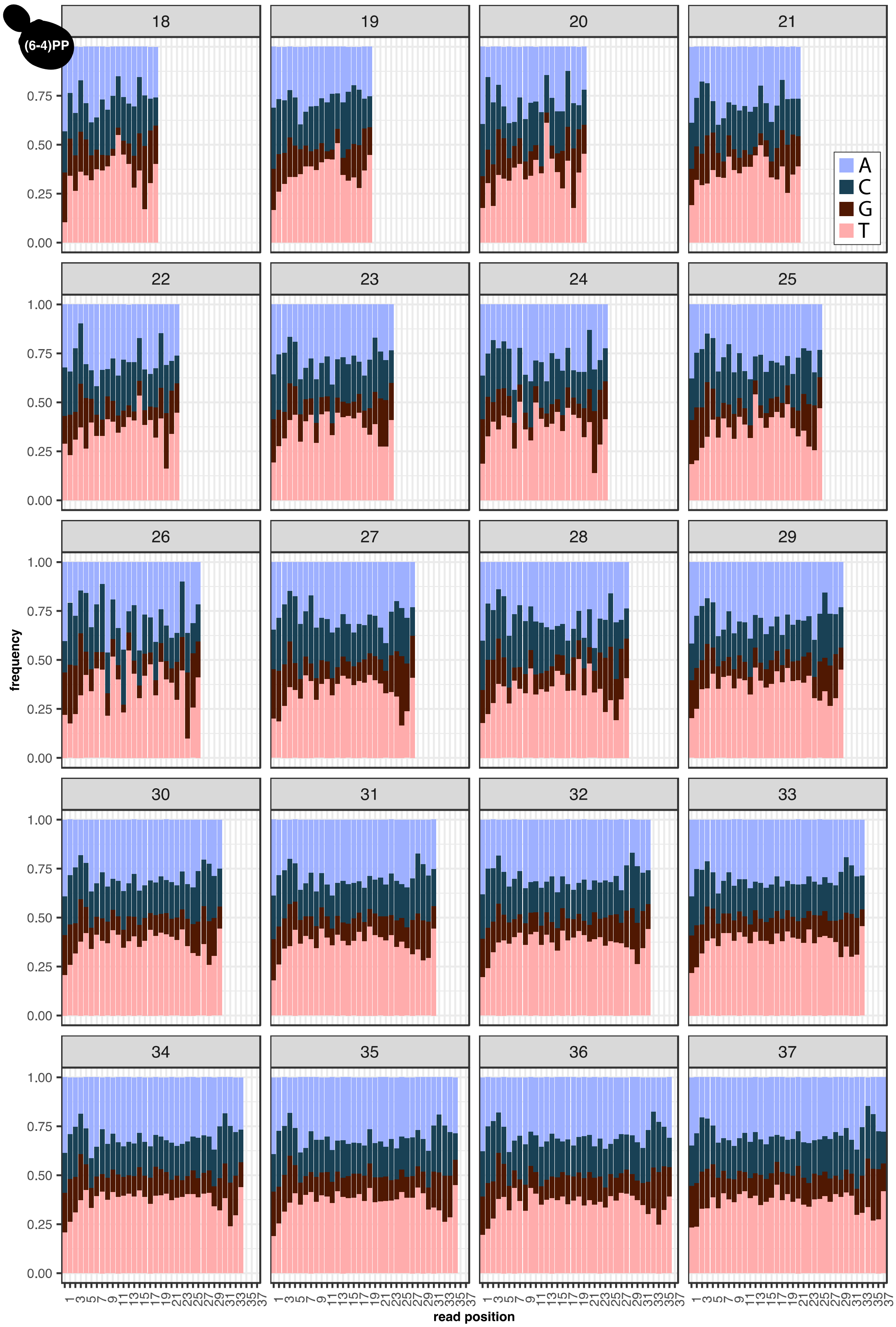

Figure S9. Nucleotide frequencies in the mtDNA-mapping reads from the *S. cerevisiae* anti-(6-4)PP libraries for all read-length classes.

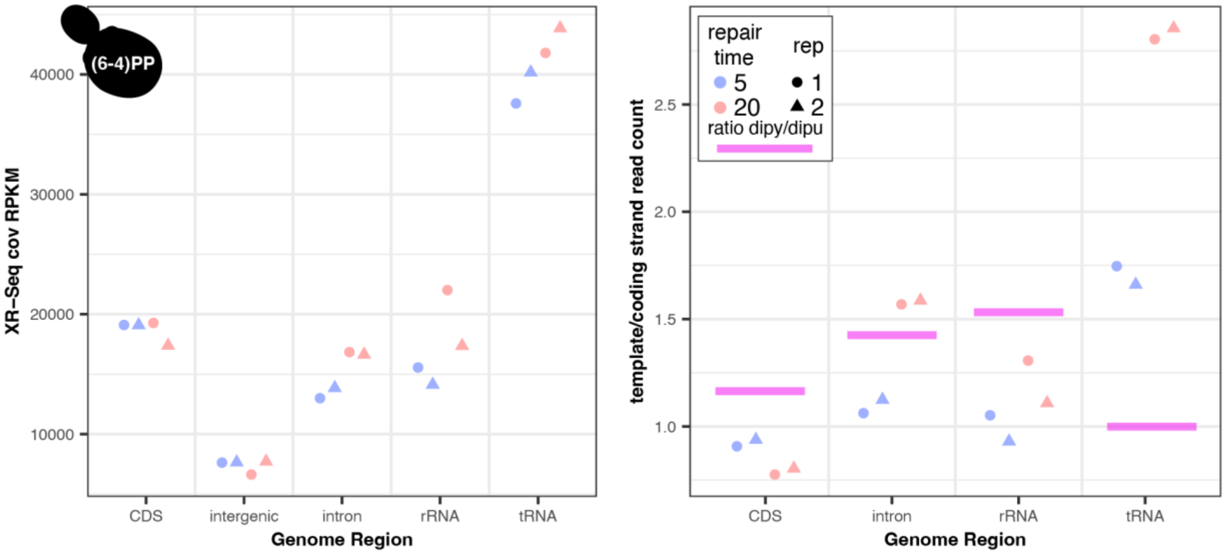

Figure S10. Depth of coverage (RPKM) and strand assymetry by genomic region in the mtDNA-mapping reads from the *S. cerevisiae* anti-(64)PP libraries. The pink bars show the ratio of dipyrimidines over dipurines on the coding strand, serving as an approximate null hypothesis for the ratio of XR-seq reads mapping to the template or coding strand.

***A. thaliana* anti-CPD libraries**

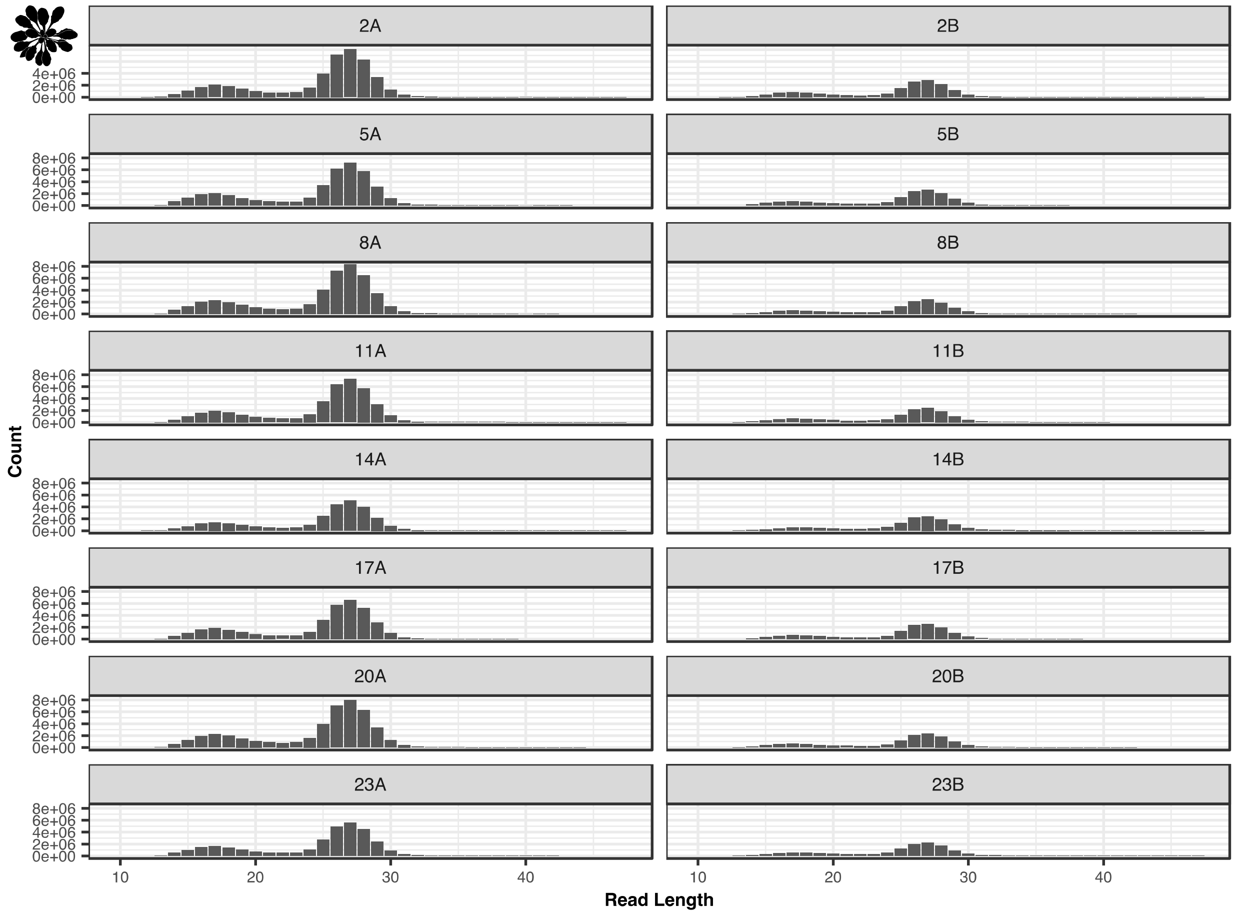

Figure S11. Length distributions of the nuclear-mapping reads from the *A. thaliana* anti-CPD libraries. The panel labels show the library ID, with the number indicating the time of day the plants were irradiated (2, 5, 8, 11, 14, 17, 20 or 23 hours) and the letter indicating the replicate (A or B).

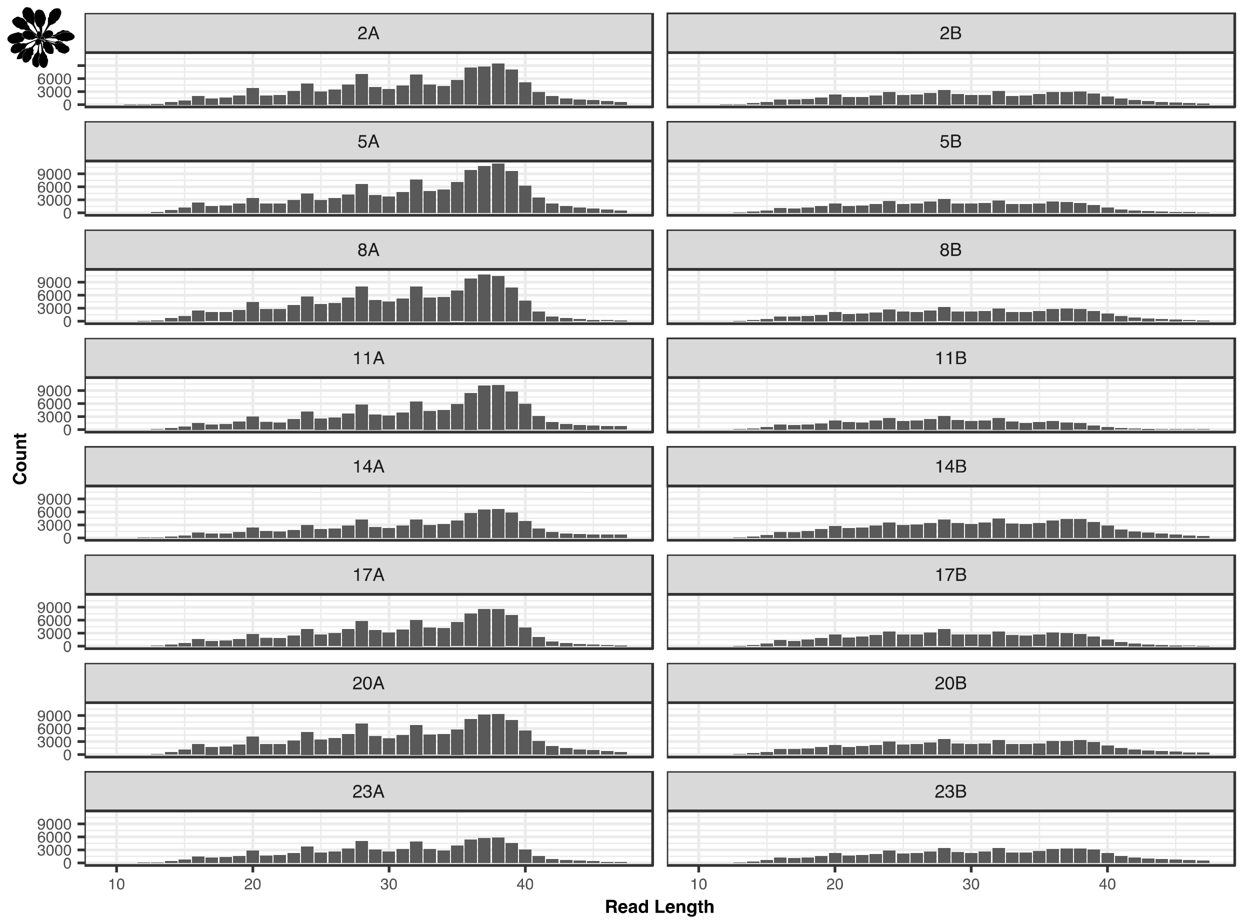

Figure S12. Length distributions of the mtDNA-mapping reads from the *A. thaliana* anti-CPD libraries. The panel labels show the library ID, with the number indicating the time of day the plants were irradiated (2, 5, 8, 11, 14, 17, 20 or 23 hours) and the letter indicating the replicate (A or B).

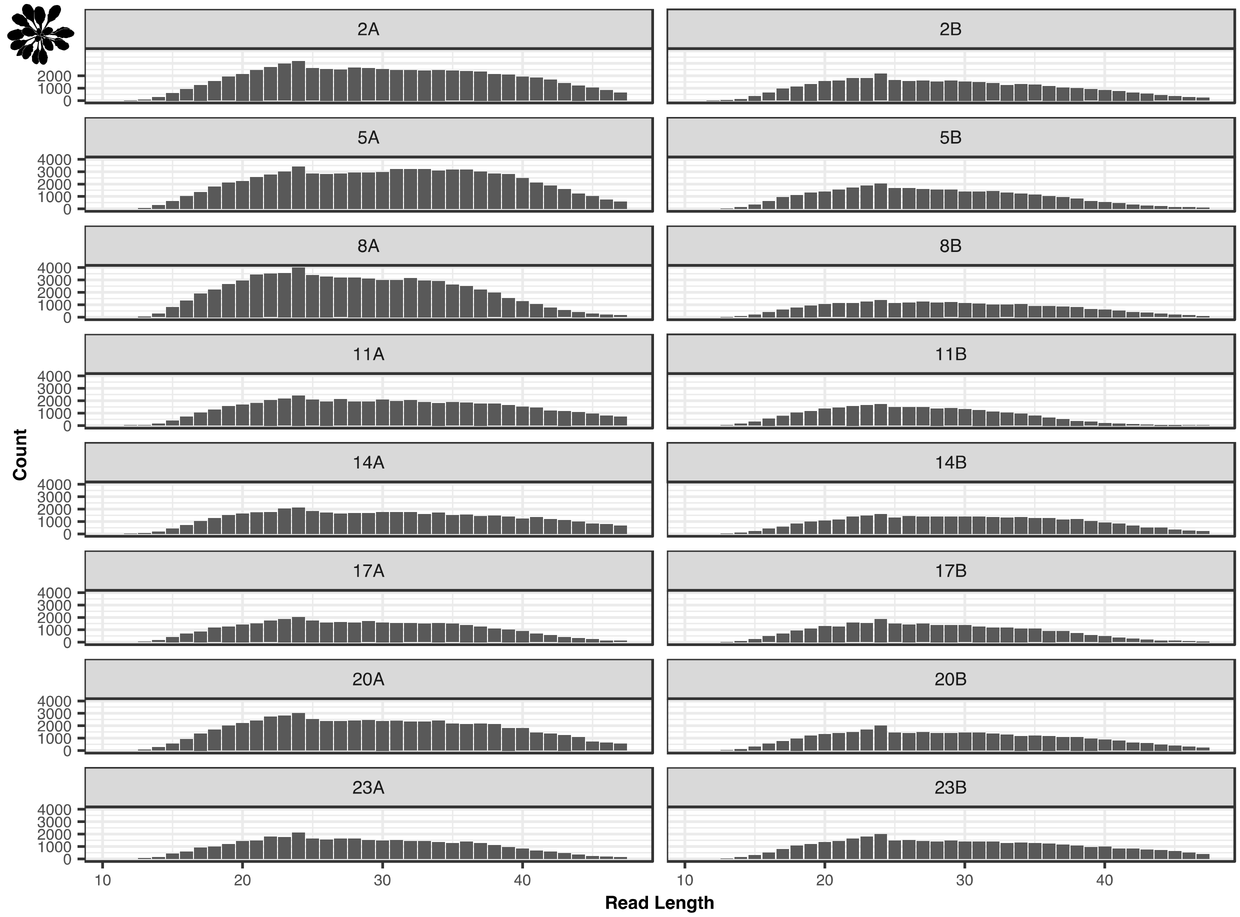

Figure S13. Length distributions of the ptDNA-mapping reads from the *A. thaliana* anti-CPD libraries. The panel labels show the library ID, with the number indicating the time of day the plants were irradiated (2, 5, 8, 11, 14, 17, 20 or 23 hours) and the letter indicating the replicate (A or B).

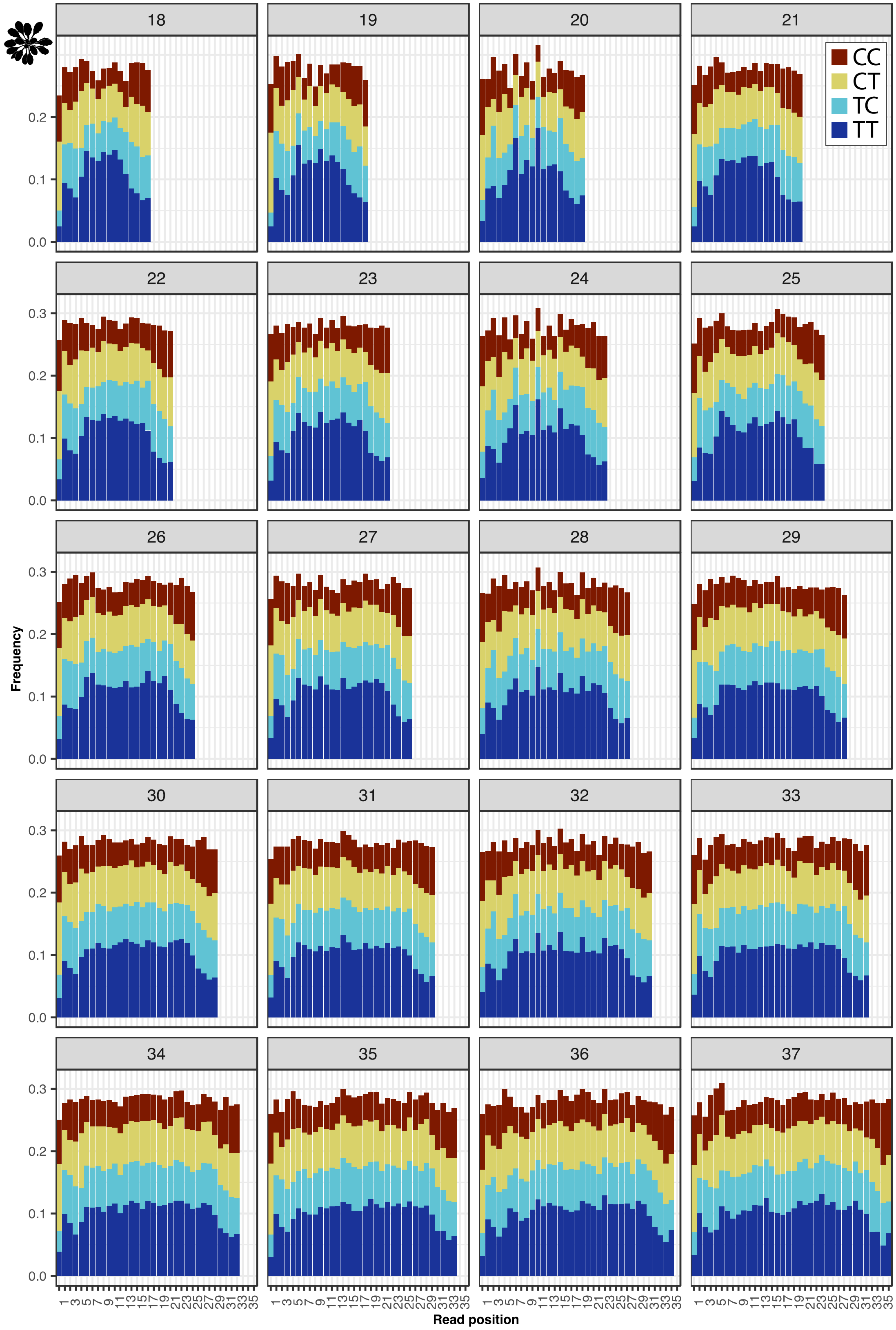

Figure S14. Di-pyrimidine frequencies in the mtDNA-mapping reads from the *A. thaliana* anti-CPD libraries.

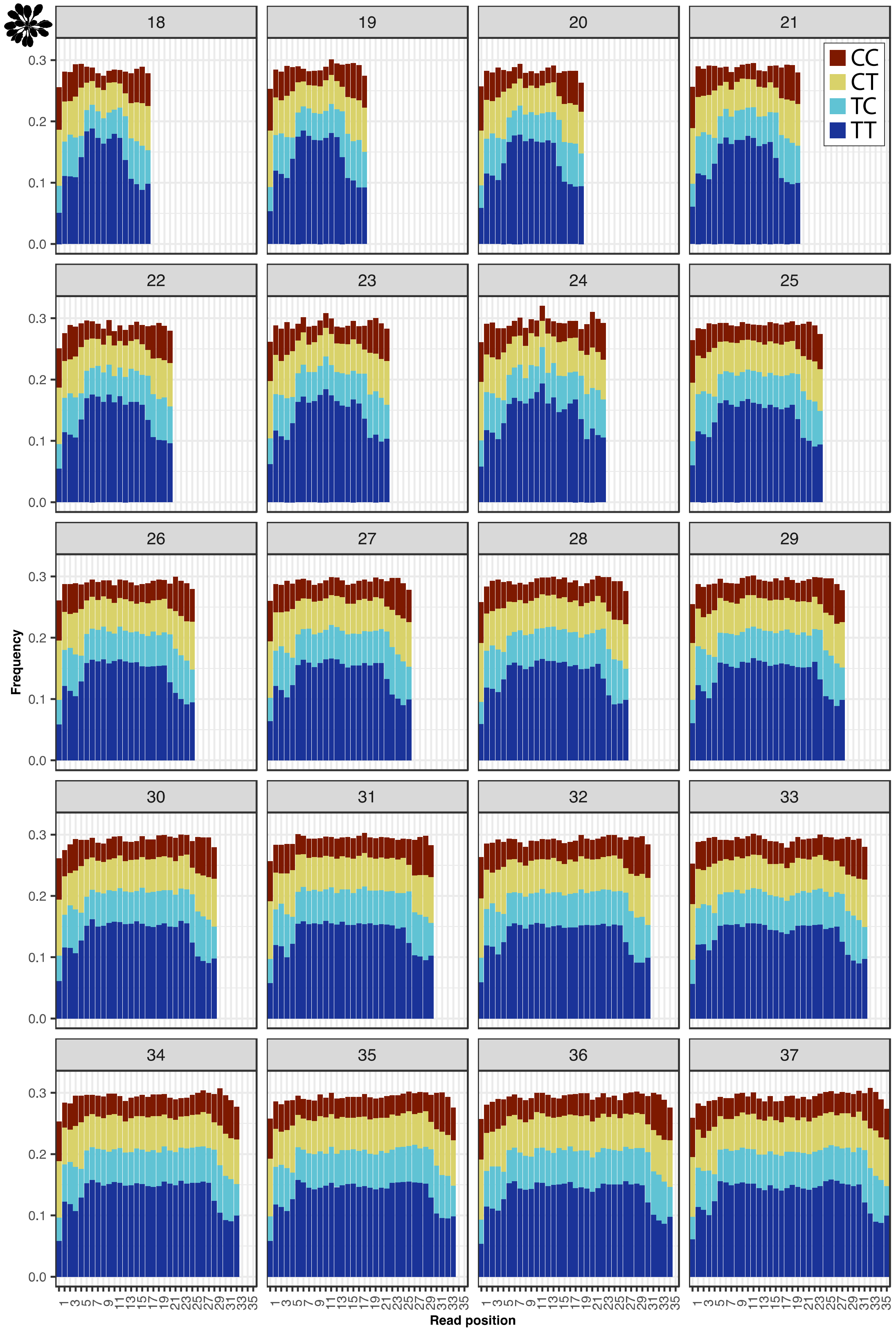

Figure S15. Di-pyrimidine frequencies in the ptDNA-mapping reads from the *A. thaliana* anti-CPD libraries.

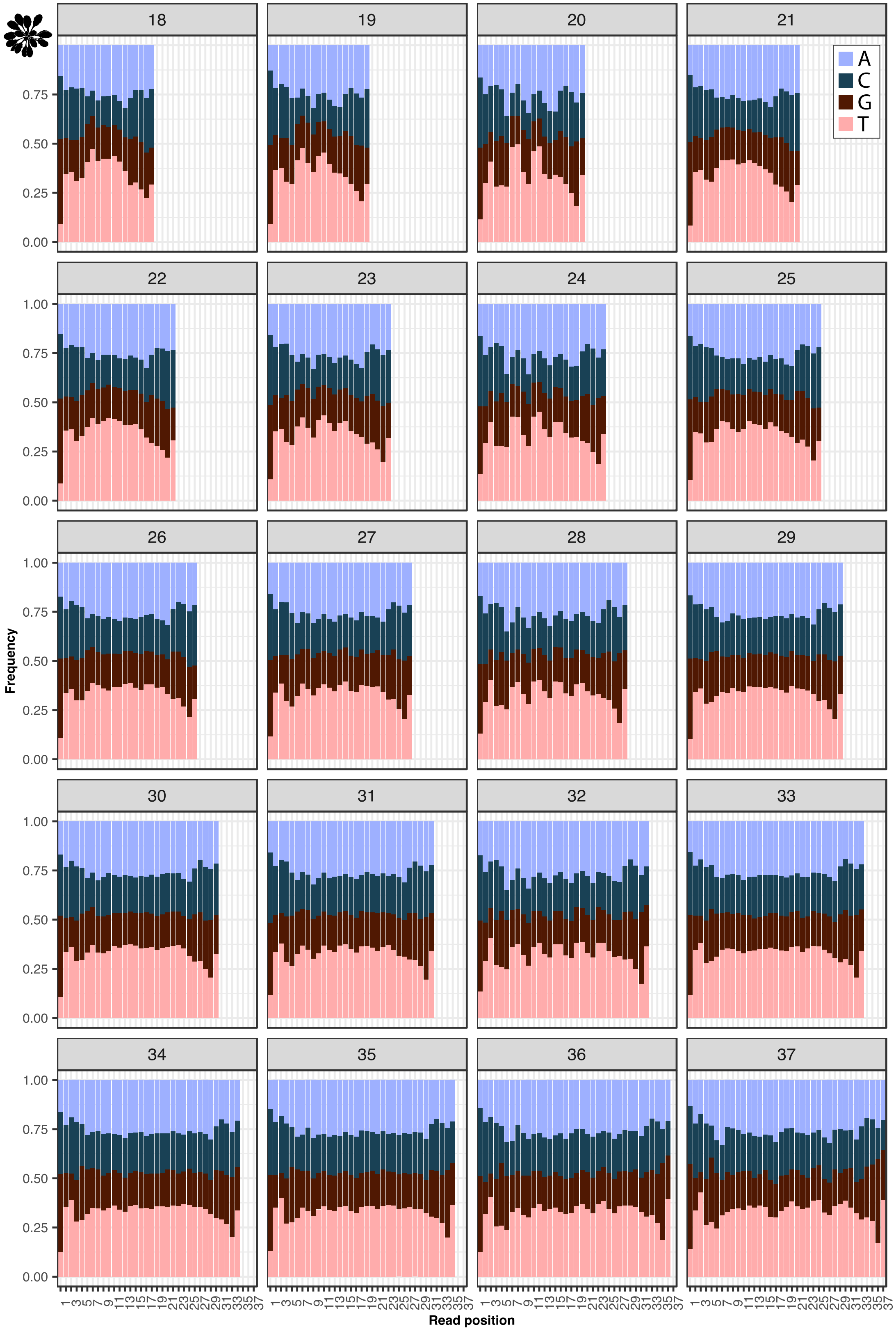

Figure S16. Nucleotide frequencies in the mtDNA-mapping reads from the *A. thaliana* anti-CPD libraries for all read-length classes.

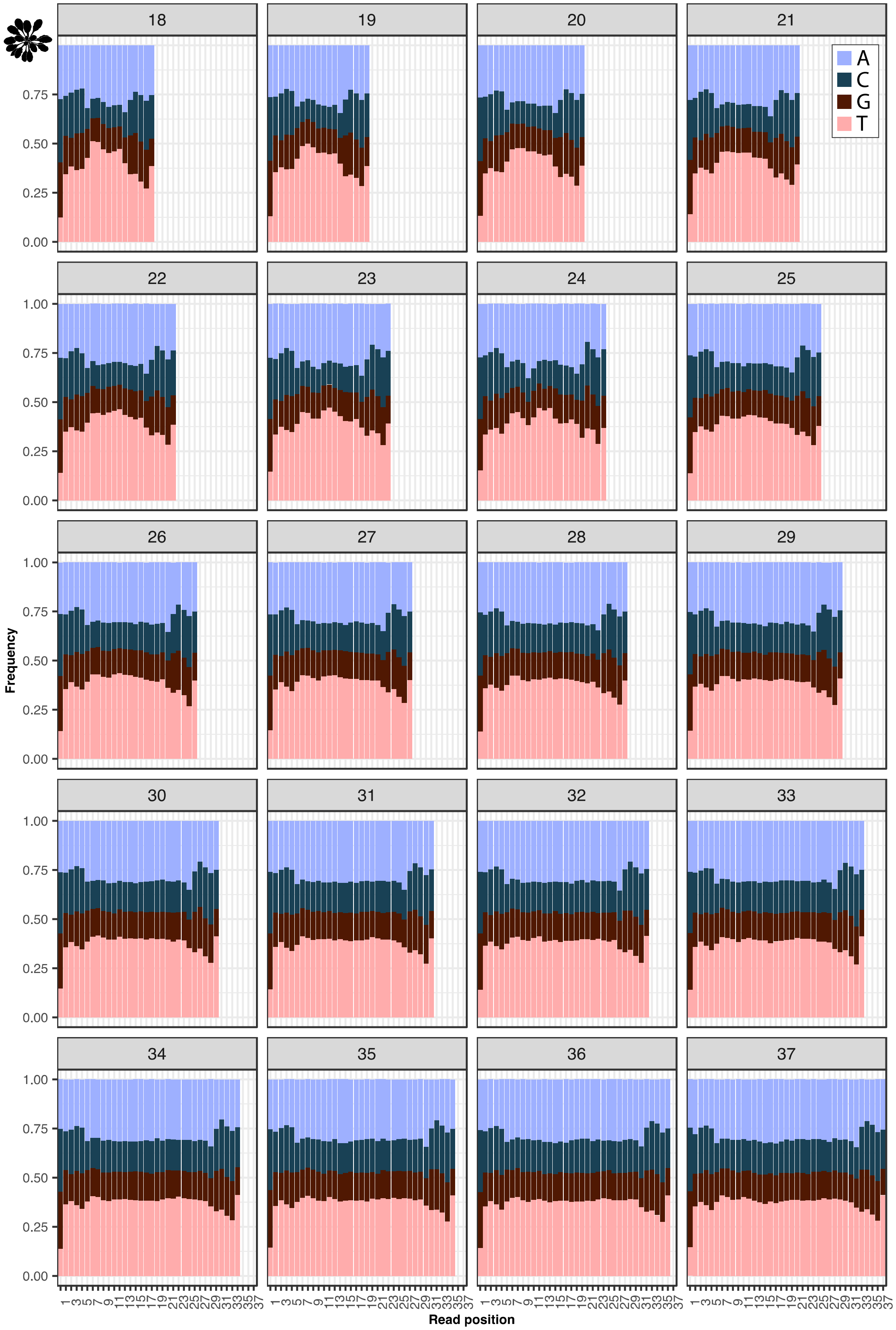

Figure S17. Nucleotide frequencies in the ptDNA-mapping reads from the *A. thaliana* anti-CPD libraries for all read-length classes.

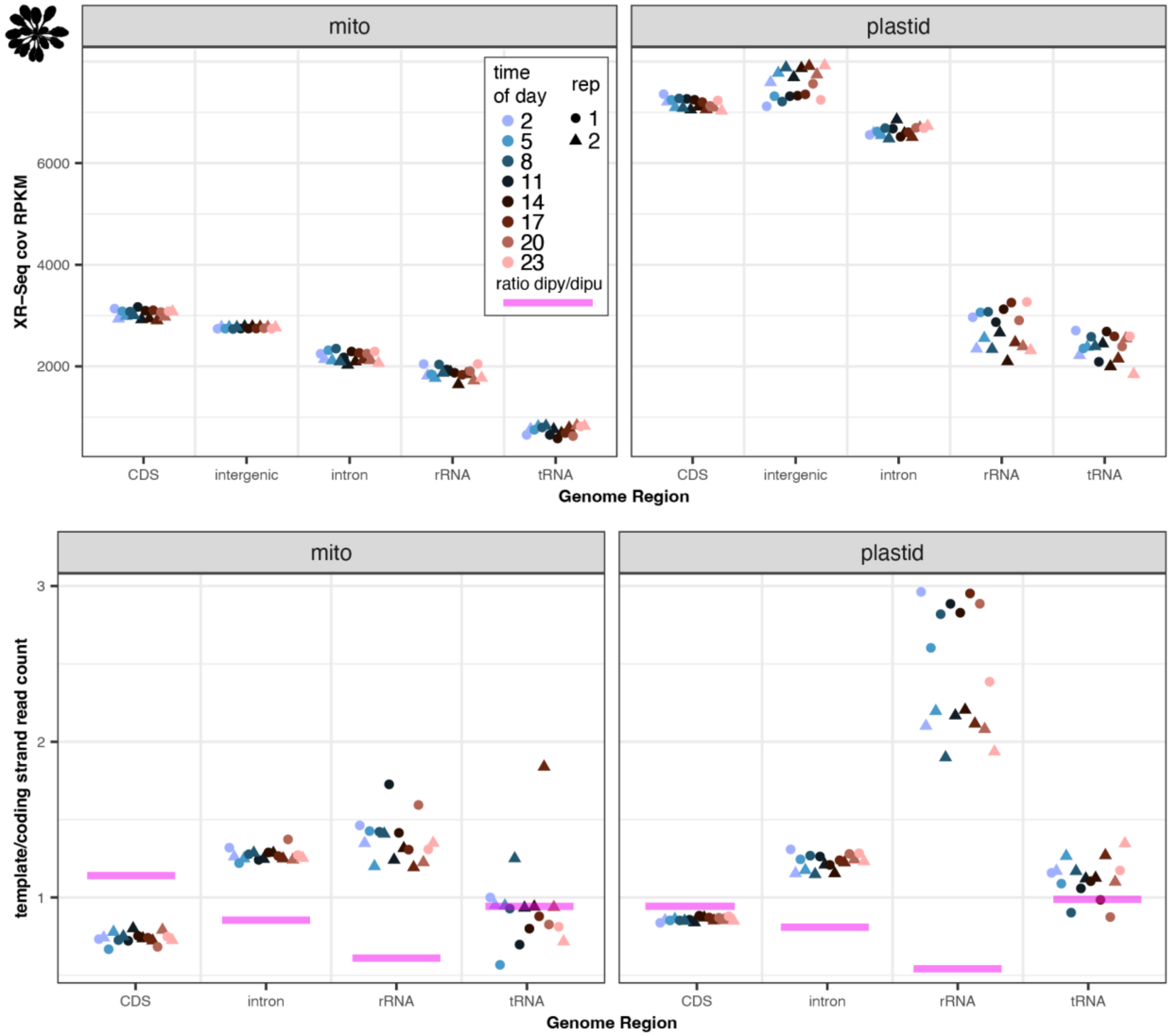

Figure S18. Depth of coverage (RPKM) and strand assymetry by genomic region in the mtDNA- and ptDNA-mapping reads from the *A. thaliana* anti-CPD libraries. The pink bars show the ratio of dipyrimidines over dipurines on the coding strand, serving as an approximate null hypothesis for the ratio of XR-seq reads mapping to the template or coding strand.

***D. melanogaster* anti-CPD libraries**

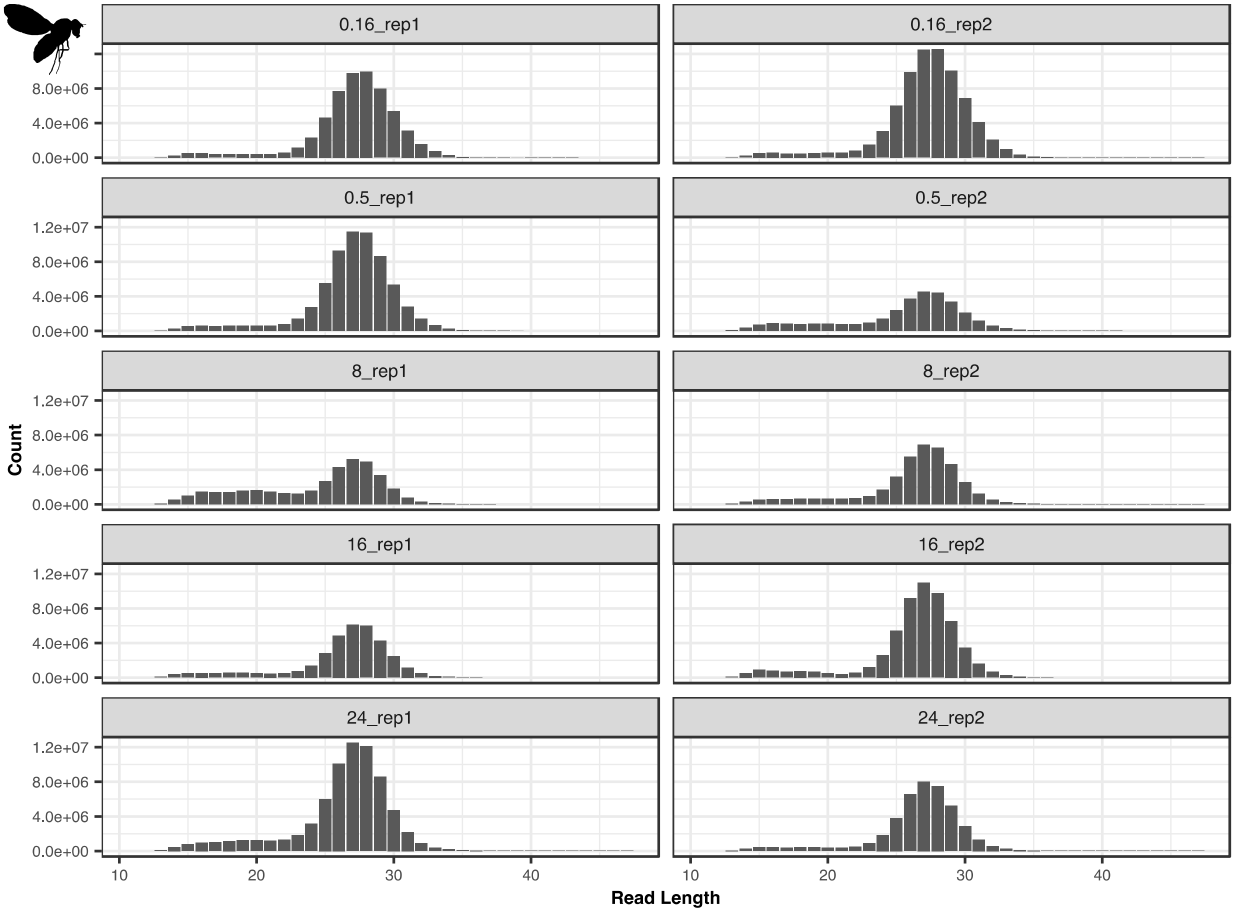

Figure S19. Length distributions of the nuclear-mapping reads from the *D. melanogaster* anti-CPD libraries. The panel labels show the library ID, with the number indicating the repair time (0.16, 0.5, 8, 16, or 24 hours) and the rep# indicating the replicate (1 or 2).

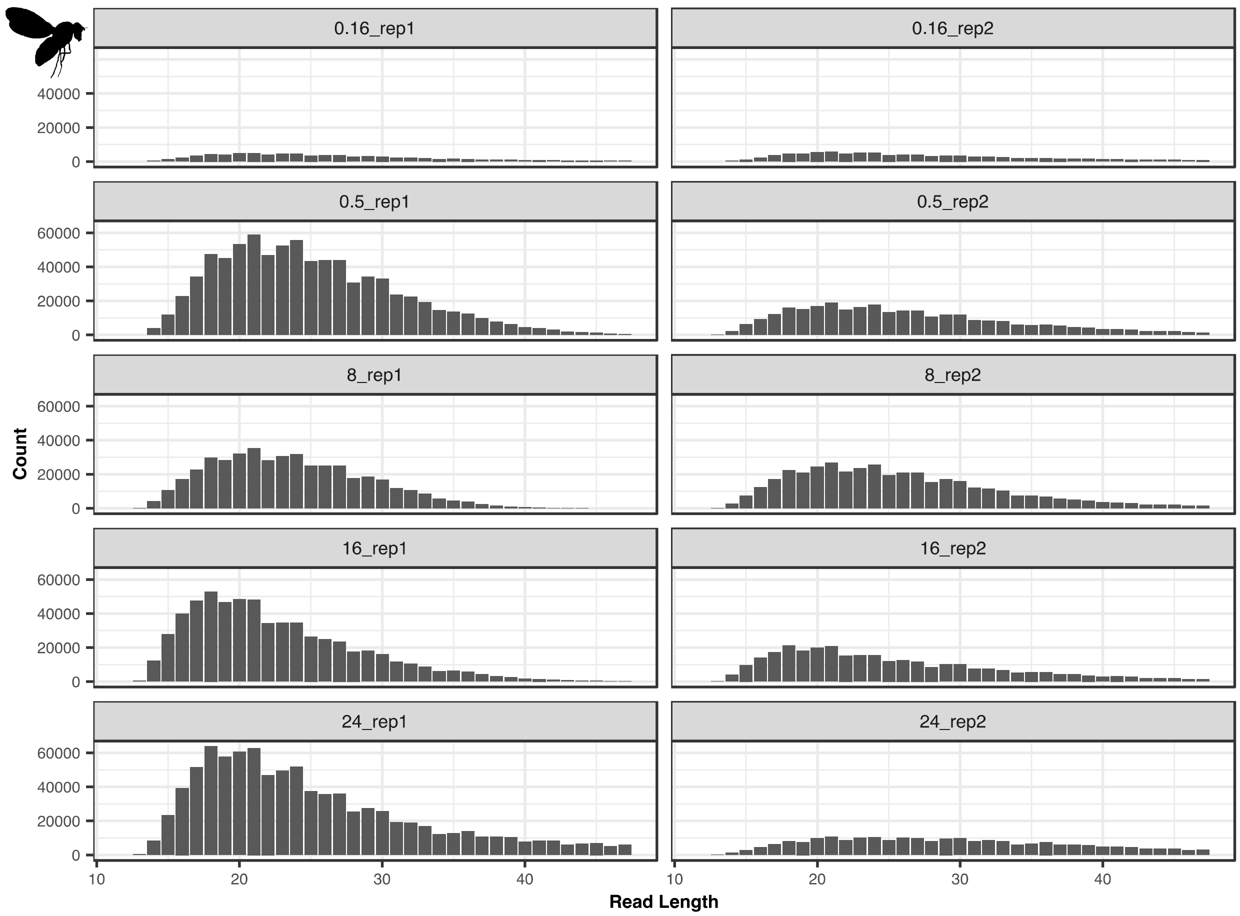

Figure S20. Length distributions of the mtDNA-mapping reads from the *D. melanogaster* anti-CPD libraries. The panel labels show the library ID, with the number indicating the repair time (0.16, 0.5, 8, 16, or 24 hours) and the rep# indicating the replicate (1 or 2).

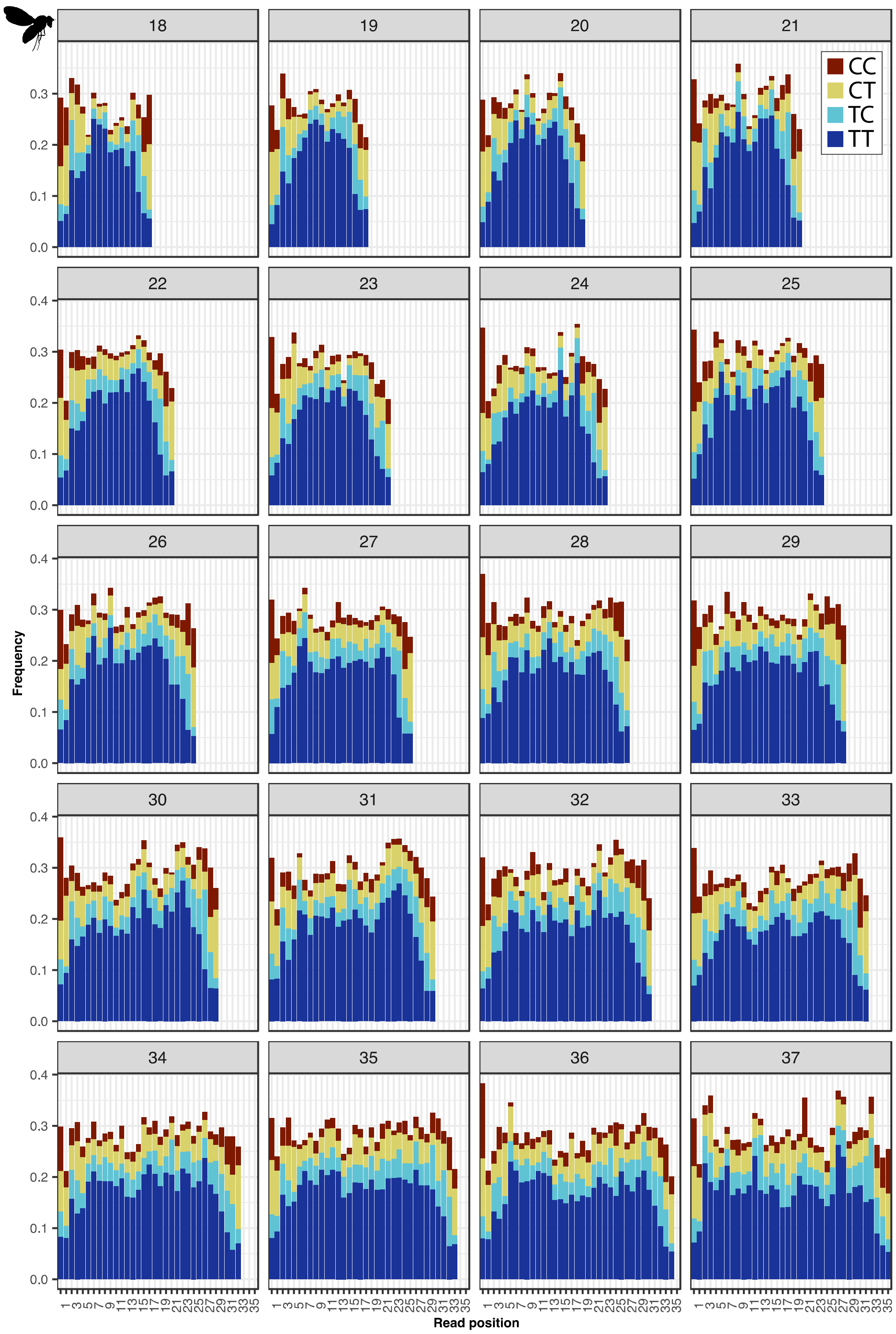

Figure S21. Di-pyrimidine frequencies in the mtDNA-mapping reads from the *D. melanogaster* anti-CPD libraries for all read-length classes.

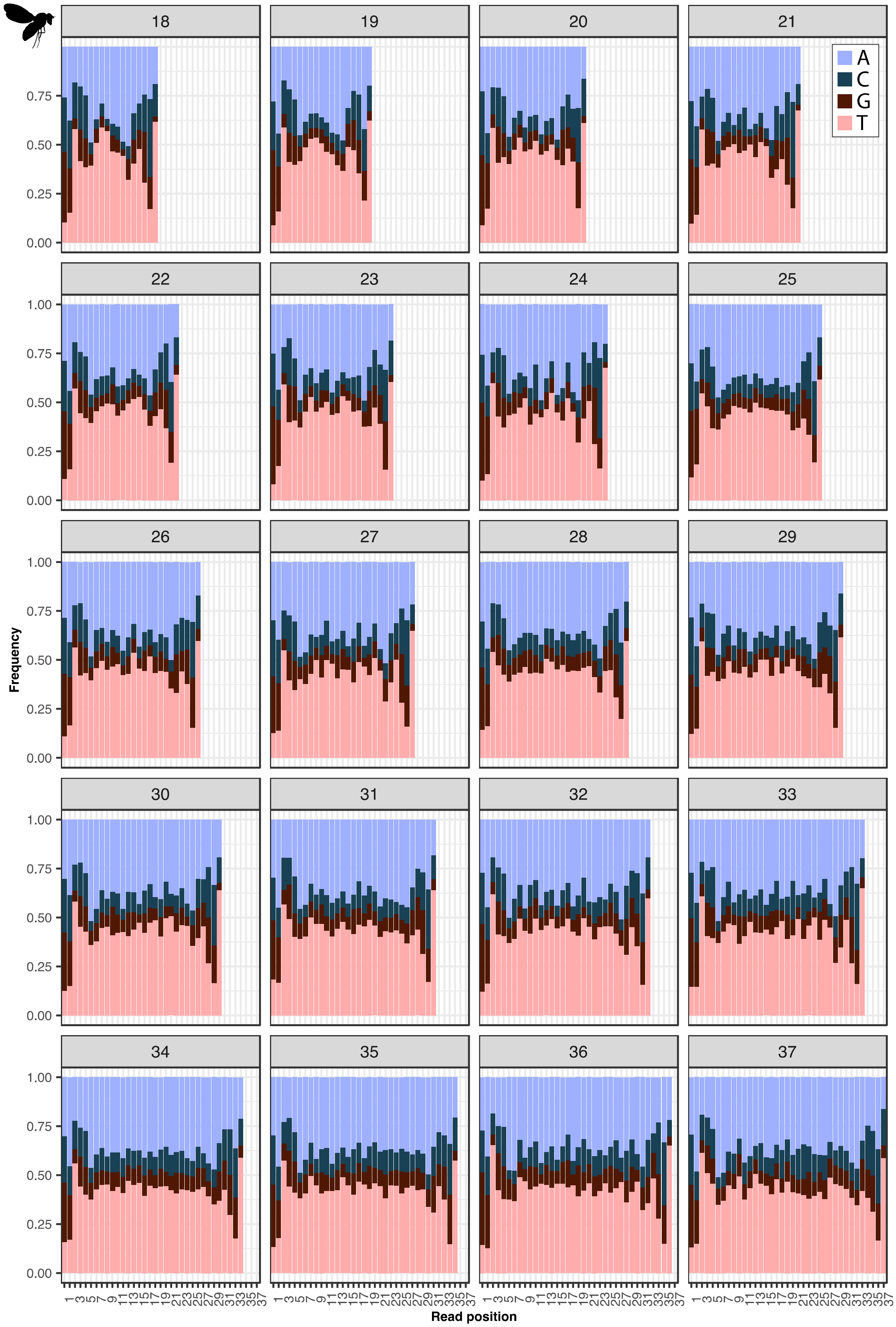

Figure S22. Nucleotide frequencies in the mtDNA mapping reads from the *D. melanogaster* anti-CPD libraries for all read-length classes.

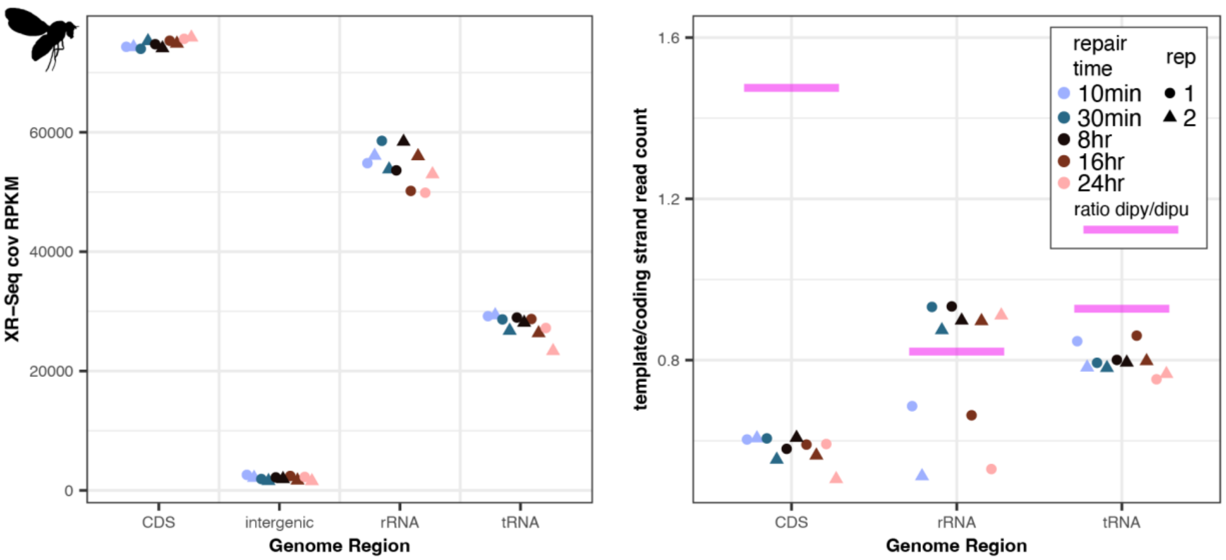

Figure S23. Depth of coverage (RPKM) and strand assymetry by genomic region in the mtDNA-mapping reads from the *D. melanogaster* anti-CPD libraries. The pink bars show the ratio of dipyrimidines over dipurines on the coding strand, serving as an approximate null hypothesis for the ratio of XR-seq reads mapping to the template or coding strand.

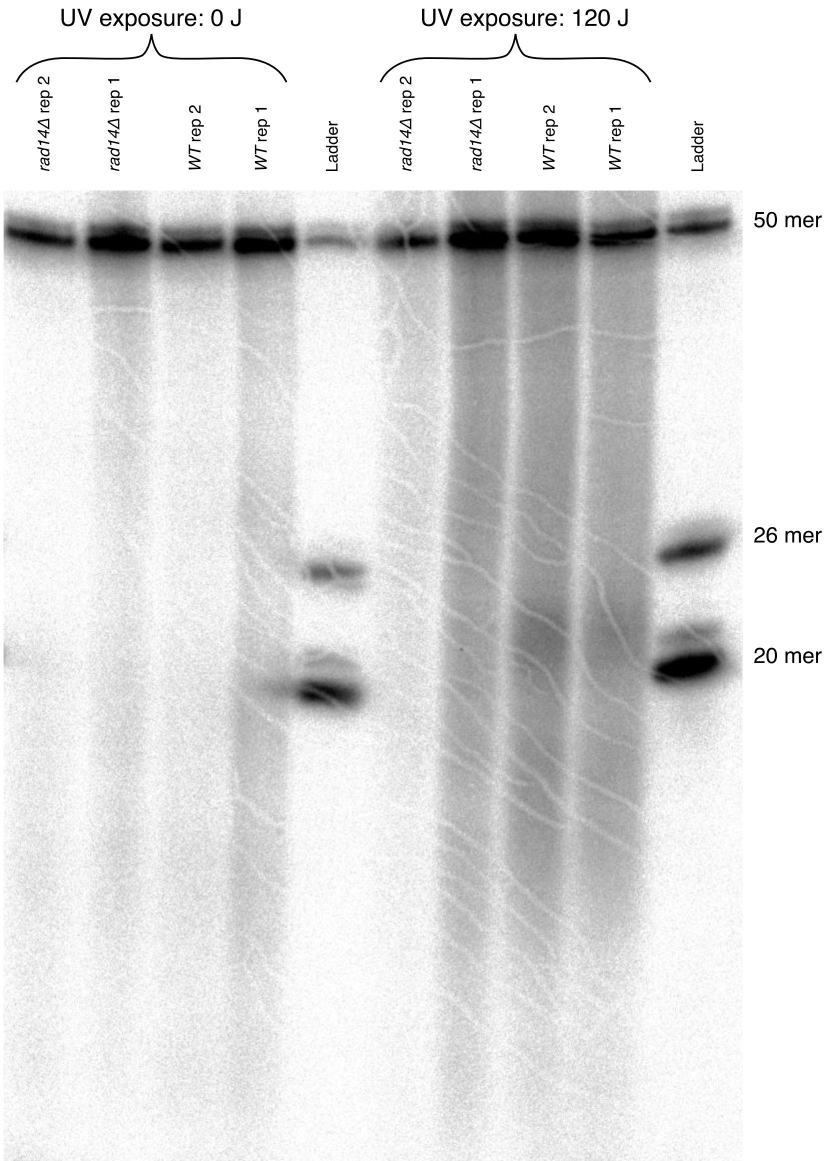

Figure S24. Excision assay gel with WT and *rad14Δ* deficient *S. cerevisiae* lines either not exposed to UV (left) or exposed to UV and given 20 minutes of dark repair (right). In conventional excision assays (Hu *et al.* 2013, 2019), 25mer and 50mer PAGE purified oligos are radiolabeled alongside samples of interest to serve as a ladder in the high density polyacrylamide electrophoresis. Under our prediction that the nuclear-NER mutant would produce only mtDNA-derived fragments, we expected to detect the 26-nt, 24-nt and 22-nt peaks that punctuate the *S. cerevisiae* read length distribution (Figure 1), and thus used PAGE purified oligos of 50, 26, and 20 nt in length to make a custom ladder. Though our 11 % acrylamide gel cracked during drying, the samples and bands can still be reliably identified. In the UV-exposed WT samples, note the darker area between the 26mer and the 20mer markers, and the slightly less dark area below the 20mer marker, corresponding to the primary products and degraded products in the excsion assay shown in Figure 1 (and Figure 2 of (Li *et al.* 2018)). We predicted to detect mtDNA-derived peaks at 26, 24 and 22 nt in the *rad14Δ* lanes, but such bands were not apparent, likely due the high background noise of this assay.
