# Supplementary material for: UV damage induces production of mitochondrial DNA fragments with specific length profiles
Source: Genetics. 2024 May 9;227(3):iyae070. doi: 10.1093/genetics/iyae070 (PMC11228841; doi:10.1093/genetics/iyae070)
Supplement: iyae070_Supplementary_Data [file iyae070_supplementary_data.zip › Supplemental_Tables_GENETICS-2024-307036.pdf]

SUPPLEMENTAL TABLES

Table S1: Primers used for generation and confirmation of *rad14Δ*

Primer Name	Sequence
JAO2397	ATTATGACTTTCTTGTTATATTCTTATATACATAACCAACAT cagatctgtttagcttgc
JAO2398	AAGAGTTTGGATCTTCGTAGTGAAGGTATCGAACGTAACGCT ggcgttagtatcgaatcg
JAO2399	ATGCACCCAAGGAATTGATTG
JAO2401	TATAGAAGCTCTATCTACAGC

The 42 nt 5'-end upper case bases in JAO2397 and JAO2398 correspond to the regions used to target the NatMX4 PCR product for integration at the *RAD14* locus to delete the gene. The 18nt 3'-end lower case bases represent the priming sequences used to amplify the NatMX4 selectable marker.

Table S2: Normalized relative abundance of di-thymines across genomic regions in *A. thaliana*, *S. cerevisiae*, and *D. melanogaster*. Absolute ratio values were calculated as the ratio of TT or AA dinucleotides divided by all other dinucleotides in each region. Normalized values were calculated by diving the region-specific absolute ratio by the genome-wide absolute ratio.

species	genome	ratio type	intergenic	CDS	intron	tRNA	rRNA
S. cerevisiae	mtDNA	absolute ratio	0.3178	0.2982	0.2794	0.2284	0.2740
S. cerevisiae	mtDNA	normalized ratio	1.0341	0.9703	0.9091	0.7431	0.8914
A. thaliana	mtDNA	absolute ratio	0.1745	0.1891	0.1487	0.1362	0.1379
A. thaliana	mtDNA	normalized ratio	1.0076	1.0916	0.8585	0.7862	0.7961
A. thaliana	cpDNA	absolute ratio	0.2959	0.2300	0.2553	0.1259	0.1138
A. thaliana	cpDNA	normalized ratio	1.1994	0.9323	1.0347	0.5104	0.4613
D. melanogaster	mtDNA	absolute ratio	0.4677	0.3188	na	0.3199	0.3826
D. melanogaster	mtDNA	normalized ratio	1.2903	0.8797	na	0.8827	1.055
